# Self‐Assembly, Aggregation Mechanisms, and Morphological Properties of Asymmetric Perylene Diimide‐based Supramolecular Polymers

**DOI:** 10.1002/chem.202501317

**Published:** 2025-07-02

**Authors:** Helal Saud M. Alharbi, Xue Fang, Robert L. Harniman, Charl F. J. Faul

**Affiliations:** ^1^ School of Chemistry University of Bristol Bristol BS8 1TS UK; ^2^ Department of Chemistry, College of Science Qassim University Buraydah 52571 Saudi Arabia

**Keywords:** mechanism, morphology, perylene diimide, self‐assembly, supramolecular polymers

## Abstract

The ability to control the self‐assembly behavior of functional molecules offers pathways for optimizing their properties and structures for targeted applications. Asymmetric perylene diimide (PDI) derivatives, bearing similar oligo(ethylene glycol) (OEG) chains and increasing numbers of hydrophobic alkyl chains were used to investigate the effects of hydrophilic and hydrophobic groups on the properties of the resulting supramolecular polymers (SMPs). Tetrahydrofuran/water (THF/H_2_O) mixtures were found to be an optimal system to induce the self‐assembly behavior of these asymmetric PDI derivatives; the behavior of these new systems was monitored by recording ultraviolet‐visible (UV/Vis) spectra and analyzing changes in absorbance intensity. Temperature‐dependent UV/Vis studies yielded thermodynamic parameters for the self‐assembly processes and showed a transition from an isodesmic to a cooperative assembly mechanism with an increase in the number of hydrophobic alkyl chains. Detailed transmission electron microscopy (TEM) and atomic force microscopy (AFM) investigations revealed the formation of nanofiber‐based supramolecular structures that exhibit a coiling tendency during their assembly process accompanied by the increase in the number (and total volume) of hydrophobic alkyl chains. Exploring these carefully tuned asymmetric PDI systems provides new opportunities to tune not only self‐assembled structures, but also mechanisms of assembly.

## Graphical abstract

Asymmetric PDI derivatives, bearing similar OEG chains and increasing numbers of hydrophobic alkyl chains were used to investigate changes in hydrophobicity on the properties of the resulting SMPs in solvent mixtures. Temperature‐dependent UV/Vis studies showed a transition from an isodesmic to a cooperative assembly mechanism with increasing hydrophobicity. Tuning molecular structure leads to tuneable self‐assembled structures and mechanisms of assembly.

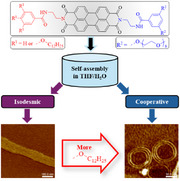

## 1 Introduction

Supramolecular polymers (SMPs) represent a unique class of materials where highly directional and reversible noncovalent interactions link the monomers. Such directed interactions between the monomers involve hydrogen bonds, π‐π interactions, metal‐ligand interactions, hydrophobic interactions, or host–guest interactions.^[^ ^1^, ^2^ ^]^ Owing to the reversibility of noncovalent interactions, SMPs exhibit dynamic behavior, enabling them to display various properties such as self‐healing, processability, and stimuli‐responsiveness.^[^ ^3^ ^]^

**Figure 1:**
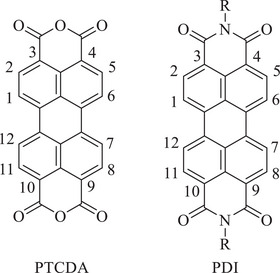
Chemical structures of PTCDA (left) and a generalized PDI (right), showing the numbering of the different positions.

Perylene diimides (PDIs), also referred to as perylene bisimides, have attracted considerable interest for their increasing prominence as building blocks in the construction of SMPs through solution‐based self‐assembly. The chemical structure of perylene‐3,4,9,10‐tetracarboxylic dianhydride (PTCDA), shown in Figure 1, can be regarded as the primary derivative of this family of compounds. PDIs feature a variety of appealing properties, including optoelectronic properties, that enable the investigation of their self‐assembly, thermal and photochemical stability, N‐type semiconductivity, and facile chemical modification.^[^ ^4^, ^5^ ^]^ These excellent properties have resulted in a diverse range of applications, including organic field‐effect transistors (OFETs),^[^ ^6^ ^]^ organic solar cells,^[^ ^7^ ^]^ light‐emitting diodes,^[^ ^8^ ^]^ and biological sensors.^[^ ^9^ ^]^

The construction of 1D self‐organized structures from PDI derivatives has gained significant research interest, mainly owing to the wide range of their potential applications.^[^ ^10^, ^11^ ^]^ The outstanding properties of PDIs enable them to be highly suitable for application in the areas of electronics, bioimaging, and photocatalysis, as illustrated by Zhu et al. and others.^[^ ^12^, ^13^, ^14^, ^15^, ^16^ ^]^ A recent study by Jiang et al.^[^ ^17^ ^]^ demonstrated the potential of PDI compounds in thermoelectric applications by highlighting their capability to readily generate anionic species as well as their remarkable material characteristics. Furthermore, these materials can be used as photosensitizers for hydrogen evolution reactions.^[^ ^17^, ^18^ ^]^

Strategic introduction of functional substituents at the imide and bay (1, 6, 7, and 12) or ortho positions (2, 5, 8, and 11) is a useful approach for designing novel functional PDIs to control the properties, self‐assembly, and thus applications.^[^ ^5^, ^19^, ^20^ ^]^ Substituents at the bay positions significantly influence the optoelectronic properties of PDI derivatives, depending on the substituent used.^[^ ^21^ ^]^ By applying particular changes to the bay positions, such as the introduction of electron‐withdrawing substituents, it is possible to alter these properties to suit specific applications, e.g., for use as active materials for OFETs.^[^ ^22^, ^23^ ^]^ The core distortion resulting from this substitution pattern, however, significantly affects the self‐assembly behavior and frequently prevents the building of well‐defined nanostructures.^[^ ^24^ ^]^ In contract, imide substitutions have a negligible effect on the optoelectronic properties of PDIs owing to the presence of molecular orbital nodes at the imide nitrogen positions.^[^ ^25^ ^]^ Such substitution can be utilized to control the self‐assembly processes, the solubility of PDI derivatives, and the morphologies of the resulting SMPs in a facile fashion.^[^ ^13^, ^26^ ^]^

Asymmetric PDIs, which bear unique substituents at each imide position, are considered an essential subgroup within PDI derivatives owing to their substantial synthetic versatility.^[^ ^27^ ^]^ Introducing a branched alkyl group at one imide position can provide high solubility, while a different substituent at the other imide position can be employed to control assembly, facilitate the formation of more complex structures, or tune functionality.^[^ ^28^ ^]^ Unlike symmetric PDIs, asymmetric PDIs are often more challenging to synthesize and purify. However, owing to the opportunities afforded by such asymmetric functionalization, this class of PDIs have become attractive when considering the inclusion of multifunctional groups to enhance π‐π stacking, electrostatic and hydrogen‐bonding interactions.^[^ ^29^ ^]^

**Figure 2:**
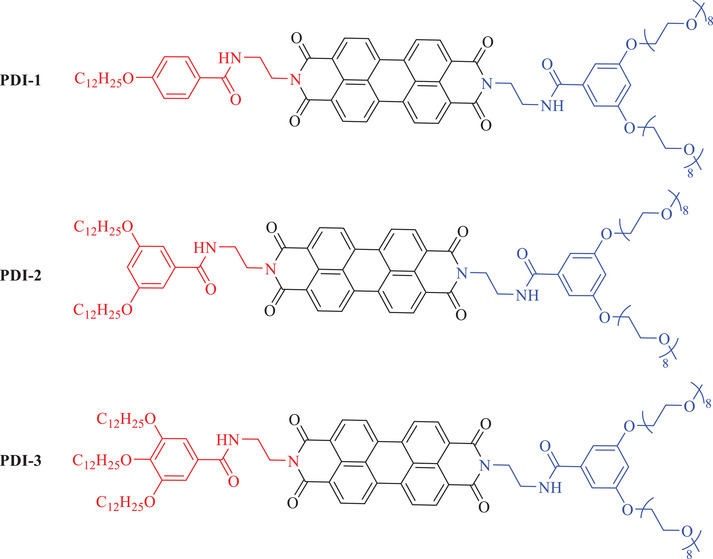
Structures of three asymmetric PDI derivatives, **PDI‐1**, **PDI‐2**, and **PDI‐3** used in this study.

Despite the promising properties of asymmetric PDI derivatives, there have been limited studies on tuning their self‐assembly behavior and morphology, especially in terms of variation of the hydrophobic‐hydrophilic balance by introducing asymmetric hydrophilic and hydrophobic chains. In this work, we aim to explore the formation of SMPs based on asymmetric PDIs, **PDI‐1**, **PDI‐2**, and **PDI‐3**, shown in Figure 2, and investigate the effects of variation of asymmetric hydrophilic and hydrophobic substitution (and thus volumes) on the properties of the resulting SMPs. We demonstrate the potential of controlling the solution self‐assembly behavior of our asymmetric PDI derivatives with simple modifications of the amide linker and changes in the alkyl and oligo(ethylene glycol) (OEG) chains at the imide position. To explore variations in a systematic fashion, we designed and synthesized the compounds **PDI‐1**, **PDI‐2**, and **PDI‐3**. These materials contain the same hydrophilic OEG chain motif, but with progressively increasing C_12_H_25_ moieties and increasing hydrophobic volume, as shown below.

## 2 Results and Discussion

### 2.1 Synthesis and Characterization

**Figure 3:**
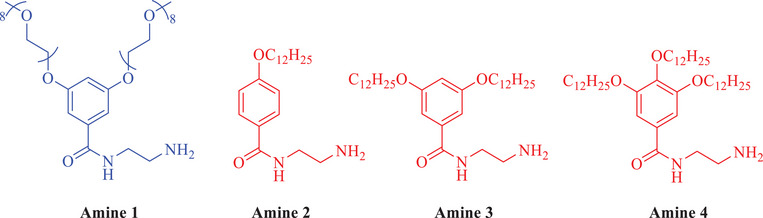
Amines employed to synthesize asymmetric PDIs featured in this work.

**Scheme 1:**
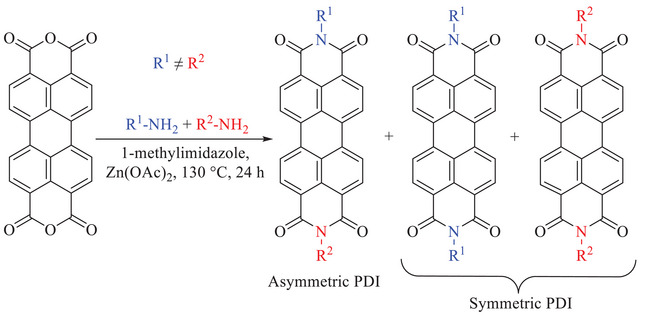
Synthetic route to produce asymmetric PDI derivatives.

Various amine substituents, as shown in Figure 3, were selected to synthesize asymmetric PDI derivatives. In all cases, the presence of the hydrogen‐bonding motif, as developed by Wuerthner et al.^[^ ^5^, ^30^ ^]^ is to ensure intermolecular hydrogen‐bonding interactions. These amines were prepared and purified according to standard reported procedures.^[^ ^31^, ^32^ ^]^ The synthesis of the desired asymmetric PDIs was accomplished by applying a one‐step technique designed by Hawker and co‐workers, which uses a stoichiometric mixture of two amines in a one‐pot reaction (Scheme 1).^[^ ^33^ ^]^ This reaction formed a mixture of symmetric and asymmetric PDI compounds; consequently, the isolation of the desired asymmetric DPI is extremely challenging. To overcome the purification challenges associated with mixtures of PDI compounds and to obtain the product, silica gel column chromatography and preparative thin layer chromatography (TLC) were extensively utilized.

Asymmetric **PDI‐1** was synthesized by reacting a mixture of **Amine 1** and **Amine 2** with PTCDA. The reaction was carried out with 1‐methylimidazole as a solvent, in the presence of zinc acetate as a catalyst at 130 °C to increase the solubility of PTCDA. Silica gel column chromatography was only effective in separating and removing the starting materials (unreacted PTCDA and amines), but not the symmetric PDIs also present in the product mixture. Thus, further purification by preparative TLC was required to separate **PDI‐1** from the two expected symmetric PDI products (Scheme 1). Matrix‐assisted laser desorption/ionization time of flight (MALDI‐ToF) spectra confirmed the successful purification of asymmetric **PDI‐1**, albeit at a reduced yield. A distribution of masses was observed in the MALDI‐ToF spectra owing to the polydisperse OEG side chains (shown in Figure S10); for example, 1656.09 *m*/*z* [M + Na]^+^ represents the mass that corresponds to OEG *DP*_n_ = 8. Following a similar method, **PDI‐2** and **PDI‐3** were synthesized, purified, and characterized. See the Supporting Information for detailed information on the synthesis and characterization by ^1^H NMR, ESI, and MALDI‐ToF mass spectroscopy. Yields obtained were 7% (**PDI‐1**), 16% (**PDI‐2**), and 15% (**PDI‐3**), respectively.

### 2.2 Solution Self‐Assembly in Tetrahydrofuran/Water Mixtures

**Figure 4:**
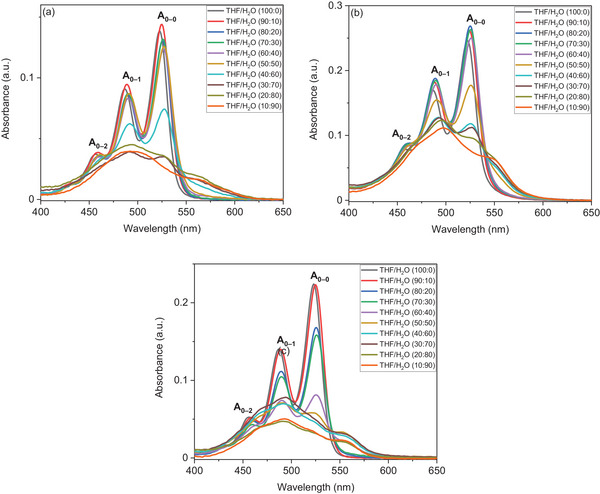
UV/Vis absorbance spectra of a) **PDI‐1**, b) **PDI‐2**, and c) **PDI‐3** recorded in THF/H_2_O mixtures at a concentration of 5 × 10^−6^ M.

To study differences in self‐assembly behavior based on the regular variation in the asymmetric structures, the PDI derivates were dissolved in tetrahydrofuran/water (THF/H_2_O) mixtures in different volumetric ratios. This solvent mixture has been demonstrated to effectively induce the aggregation of amphiphilic PDIs and hence acts as an excellent medium for revealing morphological differences.^[^ ^34^, ^35^ ^]^ In THF/H₂O mixtures, the closed‐loop miscibility gap describes the phenomenon in which the system is completely miscible at both low and high temperatures, but undergoes phase separation within a temperature range, typically between 60 and 145 °C.^[^ ^36^ ^]^ Such phase separation can influence the self‐assembly processes and overall behavior of solutes in these mixtures. However, it is important to note that our study was conducted under experimental conditions that lie outside this phase separation regime, ensuring that the observed self‐assembly behavior of asymmetric PDIs occurs within a miscible mixture. Standard procedures were applied to investigate the solution self‐assembly of asymmetric PDIs: typically, the asymmetric PDI monomer is dissolved in the chosen THF/H_2_O mixture, heated to 50 °C for 1 hour, and then cooled to room temperature and aged 24 hours prior to spectroscopic analysis. The UV/Vis spectra of **PDI‐1**, **PDI‐2**, and **PDI‐3** were therefore measured in a range of THF/H_2_O mixtures to explore the effect of variation of polarity of the solvents on the self‐assembly behavior of these systems,^[^ ^4^ ^]^ as shown in Figure 4. All three asymmetric PDIs exhibit typical absorption peaks corresponding to the S_0–0_, S_0–1_, and S_0–2_ transitions, which were clearly identified in the spectra recorded in THF between 400 and 550 nm. These peaks are labeled as A_0–0_, A_0–1_, and A_0–2_, as shown in Figure 4.

In pure THF, the absorbance spectrum of **PDI‐1** exhibits characteristic vibronic transitions for monomeric PDI molecules, with maximum absorbance (*λ*_max_) at 523 nm and gradually weaker peaks at shorter wavelengths (487 and 457 nm), corresponding to the S_0–0_, S_0–1_, and S_0–2_ transitions, respectively. **PDI‐2** and **PDI‐3** display spectra almost identical to **PDI‐1**, with *λ*_max_ at 522 and 523 nm, respectively. It is evident that there are no significant differences between the PDI absorption peaks in THF as imide substitutions have a negligible effect on optoelectronic properties of PDIs.^[^ ^25^ ^]^

The solution self‐assembly of asymmetric **PDI‐1** was investigated in THF/H_2_O mixtures at a concentration of 5 × 10^−6^ M to minimize concentration‐induced aggregation effects. As shown in Figure 4a, the absorbance spectra indicated that **PDI‐1** does not assemble into aggregates at high ratios of THF (from 100–60 v/v ratios), owing to the high solubility of the perylene core, hydrophobic tails, and OEG group in THF. Upon increasing the water ratio in the THF/H_2_O mixture to 50:50, the spectra showed a slight decrease in the intensity of the three characteristic peaks, with no clear aggregate shoulder peaks, typically at 550 nm, observed. By increasing the water ratio in the THF/H_2_O mixture to 40:60, the critical point of aggregation for asymmetric **PDI‐1** was observed: the spectra exhibited a significant decrease in the intensity of the characteristic peaks, and an aggregate peak appeared at 562 nm. This substantial decrease in absorption, accompanied by the appearance of the typical shoulder peak, was thus identified as the critical point of aggregation for **PDI‐1**. Beyond this point, the aggregate absorption peaks became more pronounced at THF/H_2_O ratios of 30:70, 20:80, and 10:90. This behavior may be attributed to the low solubility of the hydrophobic alkyl groups and the perylene core in water, in contrast to the hydrophilic OEG moieties, which can form multiple hydrogen bonds with water.^[^ ^37^ ^]^ At high water ratios, aggregation of **PDI‐1** results in a significant decrease in the intensity of the typical peaks, accompanied by a 30 nm bathochromic shift, indicating the formation of J‐aggregates.

Compared to **PDI‐1**, **PDI‐2** and **PDI‐3** showed similar behavior at high ratios of THF (for ratios 100–70). The critical points of aggregation were observed at THF/H_2_O ratios of 50:50 for **PDI‐2** and 40:60 for **PDI‐3**, respectively, as shown in Figure 4b, c. Furthermore, for both PDIs, the typical aggregate peaks appeared around 560 nm, representing the formation of J‐aggregated PDIs.

**Figure 5:**
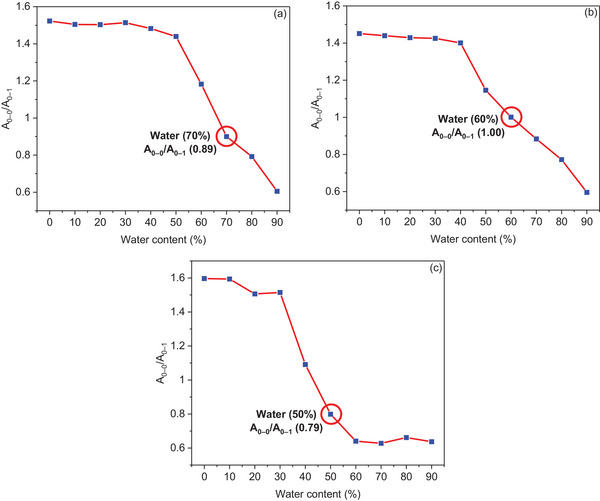
Changes in the relative intensity between A_0‐0_ and A_0‐1_ of a) **PDI‐1**, b) **PDI‐2**, and c) **PDI‐3** recorded in THF/H_2_O mixtures at a concentration of 5 × 10^−6^ M.

The intensity ratio of the S_0–0_ and S_0–1_ absorptions (A_0−1_/A_0−0_) can be employed to obtain insight into the degree of aggregation in solution.^[^ ^38^, ^39^, ^40^ ^]^ Monomeric PDI materials exhibit a characteristic Franck–Condon progression with an A_0−1_/A_0−0_ ratio of approximately 1.6. In contrast, aggregated PDIs show a decreasing ratio, reaching a limiting value of approximately 0.7.^[^ ^41^ ^]^ The A_0−1_/A_0−0_ ratios of 1.52, 1.45, and 1.56 for **PDI‐1**, **PDI‐2**, and **PDI‐3**, respectively, in pure THF, provide evidence for the presence of monomeric PDI molecules. Analysis of the A_0−1_/A_0−0_ ratios for each of the asymmetric PDIs confirms the onset of aggregation when the water ratios were increased, as seen in Figure 5. At a water ratio of 90%, these values significantly decreased to 0.60, 0.59, and 0.63, respectively, indicating strong aggregation of the PDI molecules.^[^ ^41^ ^]^ Aggregation is most likely caused by the amphiphilic nature of asymmetric PDIs; the hydrophobic perylene core and alkyl groups are poorly solubilized by the increasingly polar solvent, thereby favoring stacking to maximize hydrophobic and π–π interactions.^[^ ^25^, ^42^ ^]^ The hydrophilic OEG moieties stabilize the supramolecular polymers in the THF/H_2_O mixtures with higher water content by forming numerous hydrogen‐bonding interactions.^[^ ^31^, ^37^ ^]^

The next step in this study was to investigate the aggregation mechanism of synthesized asymmetric PDIs. Further research therefore focused on the region just beyond the critical point of aggregation, as discussed above. We continued to investigate the self‐assembly mechanism of our asymmetric PDIs at THF/H_2_O ratios of 30:70 for **PDI‐1**, 40:60 for **PDI‐2**, and 50:50 for **PDI‐3**, respectively, as shown in Figure 5 (highlighted by the red circles).

### 2.3 Mechanism of Self‐Assembly

The formation of SMPs can be described by two principal mechanisms: isodesmic and cooperative growth.^[^ ^43^ ^]^ Isodesmic growth is characterized by a consistent binding constant during polymerization, where the reactivity of a monomer and a growing polymer chain remains equal. Cooperative growth occurs in two different stages: an initial nucleation step followed by elongation of the supramolecular polymer. This mechanism is distinguished by different binding constants.^[^ ^2^, ^43^ ^]^ To identify the mechanism of supramolecular polymerization, examining changes in the degree of aggregation as a function of temperature is widely used.^[^ ^44^ ^]^ In this study, we utilized data collected from variable‐temperature UV/Vis spectroscopy to investigate the impact of changes in the balance between the hydrophilic and hydrophobic substituents on the self‐assembly mechanism.

**Figure 6:**
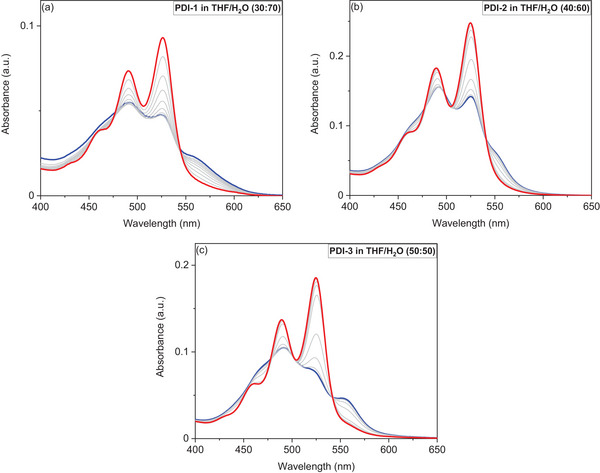
Changes in the UV/Vis absorbance spectra of **PDI‐1** a), **PDI‐2** b), and **PDI‐3** c), upon heating from 293 K (blue profiles) to 333 K (red profiles) in 5 K intervals. Spectra were recorded at a concentration of 5 × 10^−6^ M in THF/H_2_O mixtures as specified.

Changes in the absorbance profiles of all asymmetric PDIs in the THF/H_2_O mixture upon heating from 293 to 333 K are shown in Figure 6. As anticipated, all PDIs exhibited spectral changes indicative of the disassembly process with increasing temperatures. It is noteworthy that the immediate reassembly observed upon cooling from 333 K to 293 K indicate the stability of the PDI assemblies (see Figure S26–28). These investigations were repeated three times for each of the samples with no change in the UV/Vis spectra observed. These results indicate the repeatability and stability of the formed aggregates, following the same assembly mechanism for each repetition. Additional analysis of the degree of aggregation as a function of temperature for each cycle further confirms the repeatable formation and stability of the formed aggregates. **PDI‐1** and **PDI‐2** showed similar behavior in which the intensity of the aggregate peaks between 550 and 600 nm decreased gradually until the highest temperature, whereas **PDI‐3** showed a significant decrease at 313 K. Conversely, the intensity of the monomeric PDI peaks increased with rising temperatures, indicating that asymmetric PDIs exist in the form of monomers at high temperatures. By analyzing the absorption intensity data from Figure 6, the A_0−1_/A_0−0_ ratios of 0.86, 0.91, and 0.72 for **PDI‐1**, **PDI‐2**, and **PDI‐3**, respectively, at the lowest temperature provided evidence for the presence of aggregated PDI molecules.^[^ ^41^ ^]^ At the highest temperature, these values increased to 1.26, 1.35, and 1.35, respectively, which indicates the existence of monomeric PDI molecules.^[^ ^38^, ^39^, ^40^ ^]^ We again point to the highly repeatable nature of our approach, as evidenced by the presented repeated investigations (Supporting Information, Figures S26–28).

**Figure 7:**
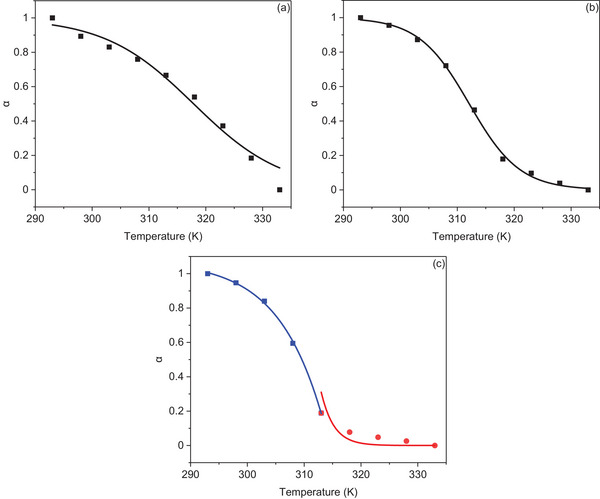
Changes in the degree of aggregation (*α*) as a function of temperature (K), fitted using either cooperative or isodesmic models: a) **PDI‐1** and b) **PDI‐2** fitted with an isodesmic model (black squares); c) **PDI‐3** fitted with a cooperative model, with data separated into the nucleation step (red circles and red line) and elongation step (blue squares and blue line).

The change in the intensity of the aggregate peak was used to determine the degree of aggregation, *α*, at each temperature for each sample using Equation S1. The factor *α* numerically represents the fraction of aggregated species in solution, where a value of 1 indicates complete aggregation and a value of 0 indicates entirely monomeric species. The degree of aggregation was then plotted as a function of temperature (K) for all PDIs, as shown in Figure 7. The data for each PDI were analyzed by attempting to fit them to either the cooperative models proposed by Smulders et al.^[^ ^44^, ^45^ ^]^ or a sigmoidal curve characteristic of the isodesmic model.^[^ ^43^ ^]^ For **PDI‐1** and **PDI‐2**, the collected data were best modeled by an isodesmic mechanism. As shown in Figures 7a, the two curves are characterized by a gradual transition from aggregated forms to monomeric species. The number average degree of polymerization (*DP*_n_) at 293 K for both PDIs was calculated using Equation S2. **PDI‐1** and **PDI‐2** exhibited *DP*_n_ values of 3.22 and 7.33, respectively, as shown in Figure S15. These values are consistent with those reported by Symons et al.^[^ ^32^ ^]^ who investigated the self‐assembly of analogous symmetrical PDI monomers with amide linkers and alkylated benzyl moieties in methylcyclohexane (MCH). In their study, PDI compounds displayed similarly low *DP*_n_ values, ranging from 3 to 7. This behavior is characteristic of isodesmic systems, wherein a significant fraction of the material tends to remain in an oligomeric state.^[^ ^43^ ^]^ The equilibrium constant (*K*_e_) was determined by applying Equation S3 to each temperature value. To obtain the thermodynamic parameters for polymerization, Van't Hoff plots, as shown in Figure S16, were used as described by Equations S4, 5. Values for changes in enthalpy (Δ*H*) were −72.44 kJ mol^−1^ for **PDI‐1**; Δ*H* increased considerably for **PDI‐2** to a value of −194.62 kJ mol^−1^. The increased enthalpic changes observed for **PDI‐2** suggest stronger intermolecular interactions within the aggregates, specifically owing to enhanced hydrophobic interactions resulting from the presence of additional alkyl chains. When compared to the values reported by Symons et al. (ranging from −78.70 to −103.50 kJ mol^−1^), it is notable that **PDI‐1** exhibited a Δ*H* value closely aligned with those findings. However, **PDI‐2** displayed a considerably larger enthalpic change, which can be attributed to the combined effect of stronger hydrophobic interactions and multiple hydrogen‐bonding interactions from the OEG moieties. A similar trend was observed in the entropy changes (Δ*S*), with a value of −141.03 J mol^−1^ K^−1^ for **PDI‐1**, while **PDI‐2** exhibited a significantly larger value of −536 J mol^−1^ K^−1^. This considerable increase in Δ*S* for **PDI‐2** further supports the presence of stronger intermolecular interactions compared with reported values. Conversely, the changes in Gibbs free energy (Δ*G*) for **PDI‐1** and **PDI‐2** are consistent with values reported in the literature, ranging from −30 to −40 kJ mol^−1^.^[^ ^32^ ^]^ These negative Δ*G* values indicate that the self‐assembly processes for both PDIs are thermodynamically spontaneous, with the aggregates forming in a favorable energy state.^[^ ^25^, ^46^ ^]^ Detailed thermodynamic parameters for **PDI‐1** and **PDI‐2** are provided in Table 1.

**Table 1:** Thermodynamic parameters for **PDI‐1** and **PDI‐2** compounds, obtained from fitting of temperature‐dependent changes in UV/Vis spectra and further analysis as described.

	Compound	
Parameter	PDI‐1	PDI‐2
*DP*_n_ (293 K)	3.22	7.33
*T*_m_ (K)	317.85	312.08
Δ*H* (kJ mol^−1^)	−72.44	−194.62
Δ*S* (J mol^−1^ K^−1^)	−141.03	−536.00
Δ*G* (kJ mol^−1^)	−31.11	−37.60

According to the analyzed data, the optimal model for **PDI‐3** was best represented by a cooperative mechanism with a nonsigmoidal sharp transition. This mechanism was initially fitted with Equation S6 for the elongation phase, followed by Equation S7 for the nucleation phase. As illustrated in Figure 7c, a distinct transition between the nucleation and elongation phases is apparent at approximately 313 K. Thermodynamic parameters obtained directly from the fitting function indicate an enthalpic change during elongation (Δ*H*_e_) of −105.19 kJ mol^−1^ and elongation temperature (*T*_e_) of 314.48 K. The calculated Δ*H*_e_ value for **PDI‐3** is closely aligned with the value reported by Ogi et al. (−108.10 kJ mol^−1^), based on the investigation of the self‐assembly of an analogous symmetric PDI monomer in a mixture of MCH and toluene at a 2:1 ratio.^[^ ^30^ ^]^ Conversely, this value increased to −137.8 kJ mol^−1^ when the system was studied in pure MCH.^[^ ^32^ ^]^ The calculated *T*_e_ value for PDI‐3 was smaller compared with literature findings, likely due to differences in the maximum temperature used in the UV/Vis studies. Jarrett‐Wilkins et al. reported the self‐assembly of an analogous PDI monomer with amide linkers and the same hydrophilic OEG moieties in an isopropanol/chloroform (iPrOH/CHCl_3_) mixture at a 9:1 ratio.^[^ ^31^ ^]^ The determined Δ*H*_e_ value was −20.9 kJ mol^−1^, which is significantly smaller than that observed for the self‐assembly of **PDI‐3** in a THF/H_2_O mixture ratio of 50:50. This comparison suggests that isopropanol cannot be classified as a poor solvent for the PDI monomer, indicating that the hydrophobic interactions among the monomer units are relatively weak within the SMPs formed in this solvent system.^[^ ^47^ ^]^ Moreover, chloroform is generally an excellent solvent for many PDI derivatives, especially those with dialkyl or diaryl imide substituents.^[^ ^26^ ^]^ In contrast, the higher polarity of water in the THF/H_2_O mixture contributes to the reduced solubility of the hydrophobic alkyl groups and the perylene core. This increased polarity will promote stronger hydrophobic and π interactions,^[^ ^48^ ^]^ thereby increasing the thermal stability of the self‐assembled structures. Equations S8, 9 were employed to estimate *DP*_n_. At 293 K, *DP*_n_ for **PDI‐3** was calculated to be 232.87. This value is consistent with those reported for analogous PDI monomers, where cooperative polymerization is typically characterized by a high degree of polymerization.^[^ ^44^ ^]^ Detailed thermodynamic parameters for cooperatively polymerized **PDI‐3** are provided in Table 2. Further details of these parameters are given in the Supporting Information.

**Table 2:** Thermodynamic parameters for **PDI‐3**, obtained from fitting of temperature‐dependent changes in UV/Vis spectra and further analysis as described.

	Compound
Parameter	PDI‐3
*N*_n_ (293 K)	232.87
*T*_e_ (K)	314.48 ± 0.17
Δ*H*_e_ (k J mol^−1^)	−105.19 ± 7.02
*K*_a_	3.28 E‐3 ± 6.10 E‐3
*N*_n_ (*T*_e_)	6.73

In our asymmetric (hydrophobic–hydrophilic) PDI‐based SMP system, a clear transition from isodesmic polymerization to cooperative polymerization was observed with the successive addition of hydrophobic C_12_ alkyl groups. These changes also significantly influenced the resultant physical properties, such as the enthalpic changes, with the PDI compounds exhibiting more negative Δ*H* values for **PDI‐2** and **PDI‐3** compared with **PDI‐1**.^[^ ^49^, ^50^ ^]^ Since these SMP systems were studied under similar conditions, this trend can be attributed to variations in the strengths of noncovalent interactions within the aggregates, particularly π–π stacking and hydrophobic interactions.^[^ ^51^, ^52^ ^]^ These interactions lead to more stable and well‐defined aggregates for **PDI‐3** compared with **PDI‐1**, resulting in the formation of supramolecular polymers with significantly higher *DP*_n_ through a highly cooperative polymerization mechanism.

### 2.4 Morphology of the Aggregates

**Figure 8:**
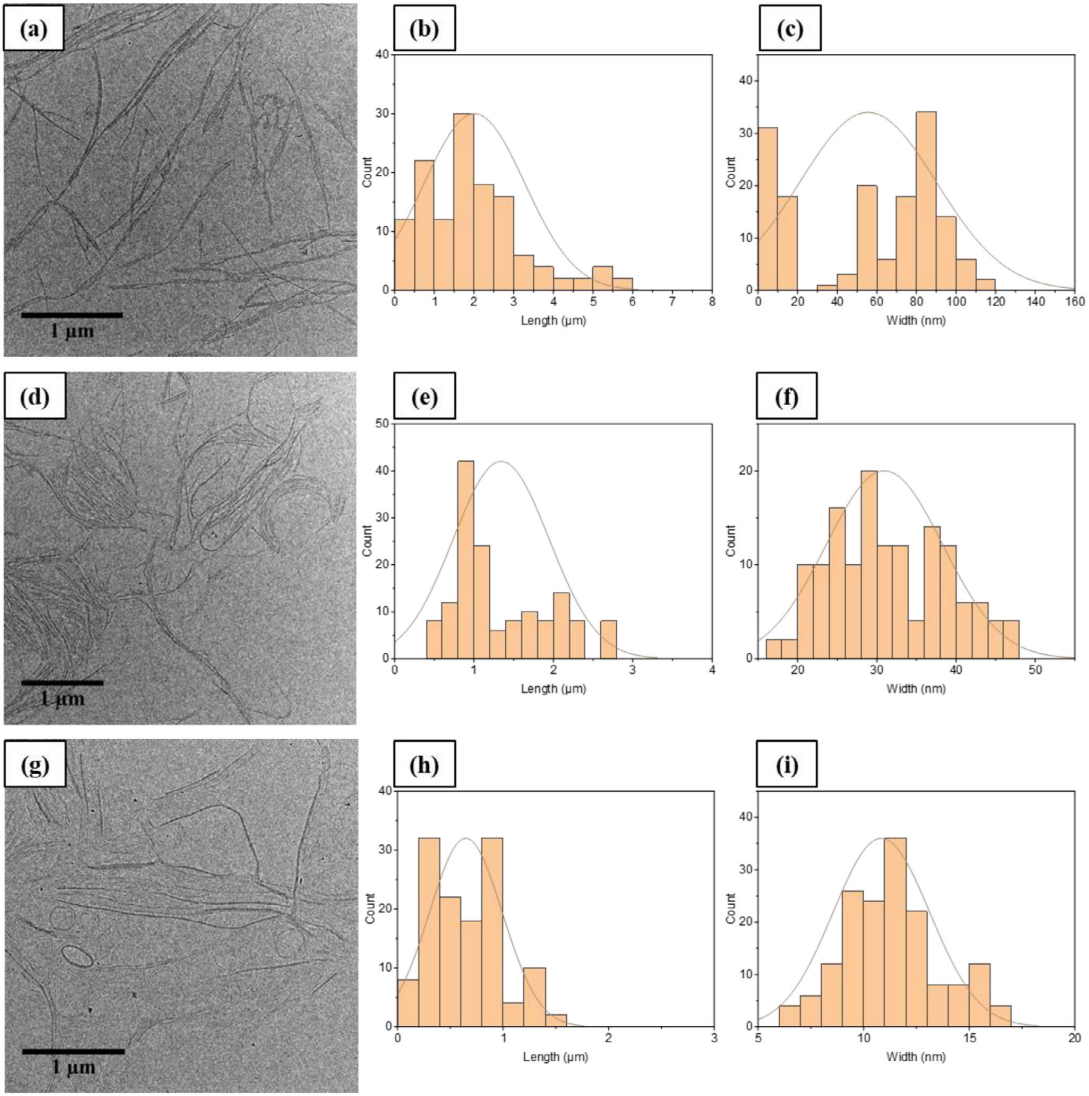
TEM images of the morphologies formed by the self‐assembly of a) **PDI‐1** in THF/H₂O (30:70), d) **PDI‐2** in THF/H₂O (40:60), and g) **PDI‐3** in THF/H₂O (50:50) at a concentration of 5 × 10^−6^ M. The corresponding length distributions are shown in b, e, and h), and the width distributions in c, f, and i).

To further explore differences in behavior amongst these synthesized PDIs, transmission electron microscopy (TEM) and atomic force microscopy (AFM) were used to characterize the morphology of the formed aggregates from THF/H_2_O mixtures. TEM images of the typical morphologies formed by each PDI in the respective selected mixtures are shown in Figure 8. Further high‐magnification TEM images for each PDI are provided in the Supporting Information (Figure S25). The TEM analysis of **PDI‐1** in THF/H_2_O (30:70) revealed two distinct stages of self‐assembly. Initially, the self‐assembly process yields thin nanofibers with widths of approximately 8 nm, with these nanofibers aggregating into ribbon‐like structures with widths ranging from 60 to 120 nm (see Figure 8a, and the width distribution histogram shown in Figure 8c). The length distribution ranged from hundreds of nanometers to several micrometers, with a mean length of 2.00 µm (shown in Figure 8b). The fibers formed by self‐assembly of **PDI‐2** (Figure 8d) showed a tendency to aggregate into partially coiled morphologies with a mean width of 30 nm (see width distribution histogram shown in Figure 8f). The length distribution ranged from hundreds of nanometers to several micrometers, but with a shorter mean length of 1.34 µm (shown in Figure 8e). Additional increases in hydrophobic alkyl chain number (and thus hydrophobic volume) significantly influence the self‐assembly behavior of the asymmetric **PDI‐3** compared with other PDIs. The observed **PDI‐3** morphologies, as shown in Figure 8g, consist of well‐organized supramolecular fibers with typical widths ranging from 5 to 15 nm and length distributions with a mean length of 0.64 µm (shown in Figure 8h), forming coiled or spiral structures.^[^ ^35^ ^]^ See Tables S1, 2 for a detailed overview of dimensions obtained from TEM and AFM investigations, respectively.

It is important to note that the nanofibers formed by all PDIs do not exhibit crystallinity. Selected area electron diffraction (SAED) analysis was conducted on a selection of samples; however, no clear diffraction patterns were observed in the data. The high‐energy electron beam used in TEM has the potential to anneal crystallinity in assembled PDI structures, which may contribute to the absence of detectable diffraction patterns. Additional SAED data are provided in the Supporting Information (see Figure S21).

The notable curvature observed in the self‐assembled fibers by TEM suggests that the fibers exhibit increased flexibility with the addition of hydrophobic alkyl chains, potentially allowing the fibers to bend during the drying process on the TEM grid.^[^ ^35^ ^]^ This flexibility may originate from the steric bulk of the asymmetrically increasing hydrophobic alkyl chains at the imide positions, which influences the packing arrangement of PDI monomers within the supramolecular polymer.^[^ ^35^, ^50^ ^]^

**Figure 9:**
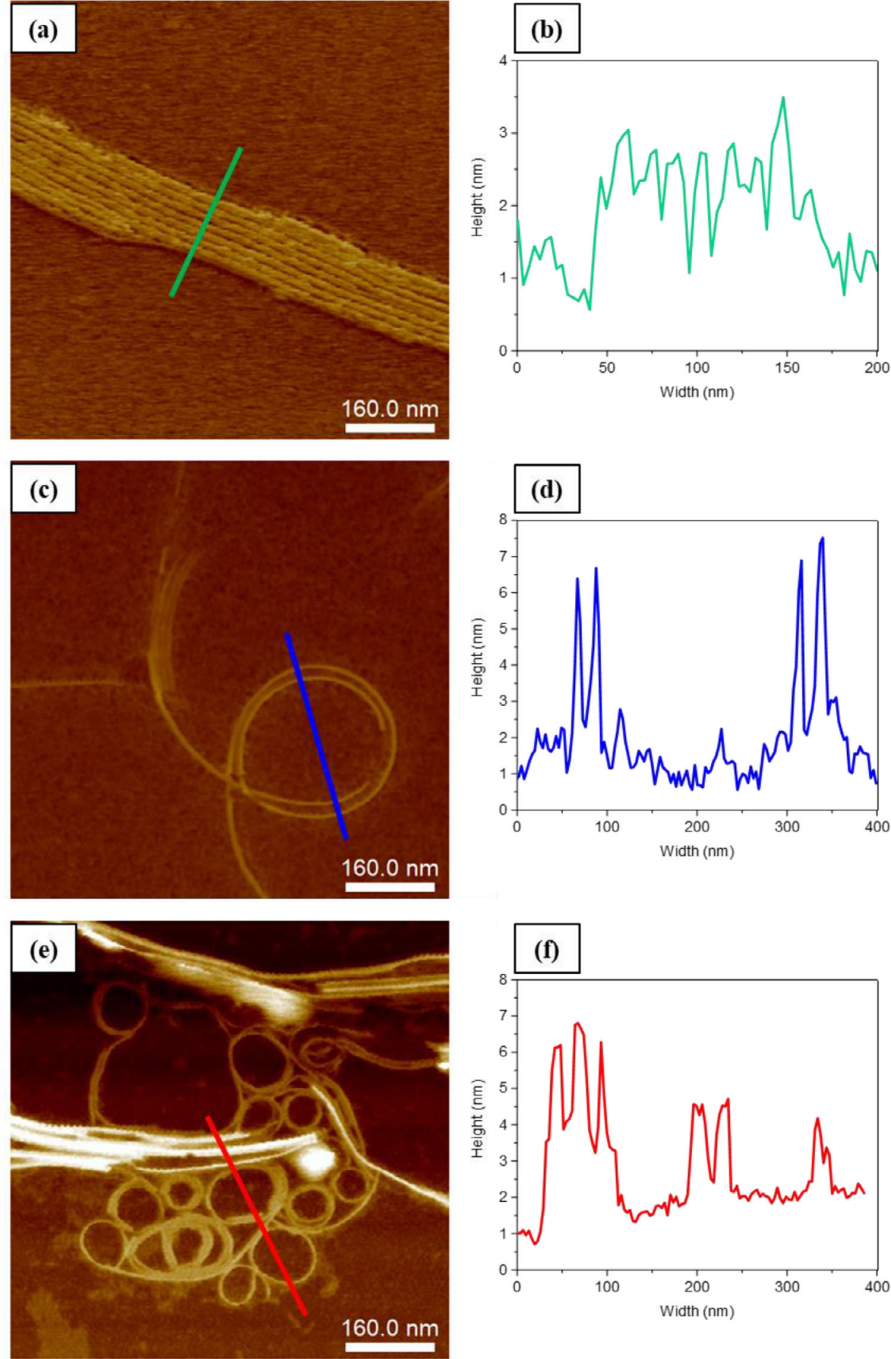
AFM images of the morphologies formed by the self‐assembly of a) **PDI‐1** in THF/H_2_O (30:70), c) **PDI‐2** in THF/H_2_O (40:60), and e) **PDI‐3** in THF/H_2_O (50:50) at a concentration of 5 × 10^−6^ M. Height profiles along the corresponding colored lines are shown in b, d, and f). The *z*‐range is 10 nm for a), 10.40 nm for c), and 12.30 nm for e).

Further investigation of PDI derivatives on mica substrates by AFM revealed similarities in the overall trends of the morphologies observed by TEM. Figure 9 shows specific AFM images of the morphologies formed by each PDI in the mixture of THF/H_2_O, where an increasing tendency for curvature is evident from **PDI‐1** to **PDI‐3**. Further typical AFM images for each PDI are provided in the Supporting Information (Figure S17). The AFM analysis of **PDI‐1** elucidates the formation of aligned supramolecular structures, characterized by typical fiber heights of 1.50 nm (see height distribution histogram shown in Figure S18). Occasionally larger, aligned structures with heights extending to approximately 3.50 nm are observed, but these are in the minority and are likely formed through aggregation. In agreement with TEM data, the initial self‐assembly of **PDI‐1** leads to the formation of individual nanofibers with widths of roughly 13 nm, as determined from the line profiles. The observed morphologies suggest that the nanofibers then likely undergo lateral aggregation, facilitated by π‐π stacking interactions and potentially enhanced by hydrophobic interactions from the alkyl chains.^[^ ^53^ ^]^

The fibers formed by **PDI‐2** self‐assembly exhibited wavy and curved structures, characterized by typical heights of approximately 2.70 nm, extending to ca. 7.80 nm through successive aggregation (see height distribution histogram in Figure S19). These defined morphologies also have typical widths of ca. 13 nm, similar to those observed by TEM. Consistent with the TEM findings, the AFM images also demonstrate that **PDI‐3** forms well‐organized supramolecular coiled fiber structures, as seen in Figure 9e. Most of these fibers exhibit typical heights of approximately 2.5 nm (height distribution histogram shown in Figure S20) and widths of 20 nm. Furthermore, clusters of nanofibers in **PDI‐3** exhibit areas with increased width and height (80 and 35 nm, respectively), as a result of the lateral aggregation of supramolecular fibers.

**Figure 10:**
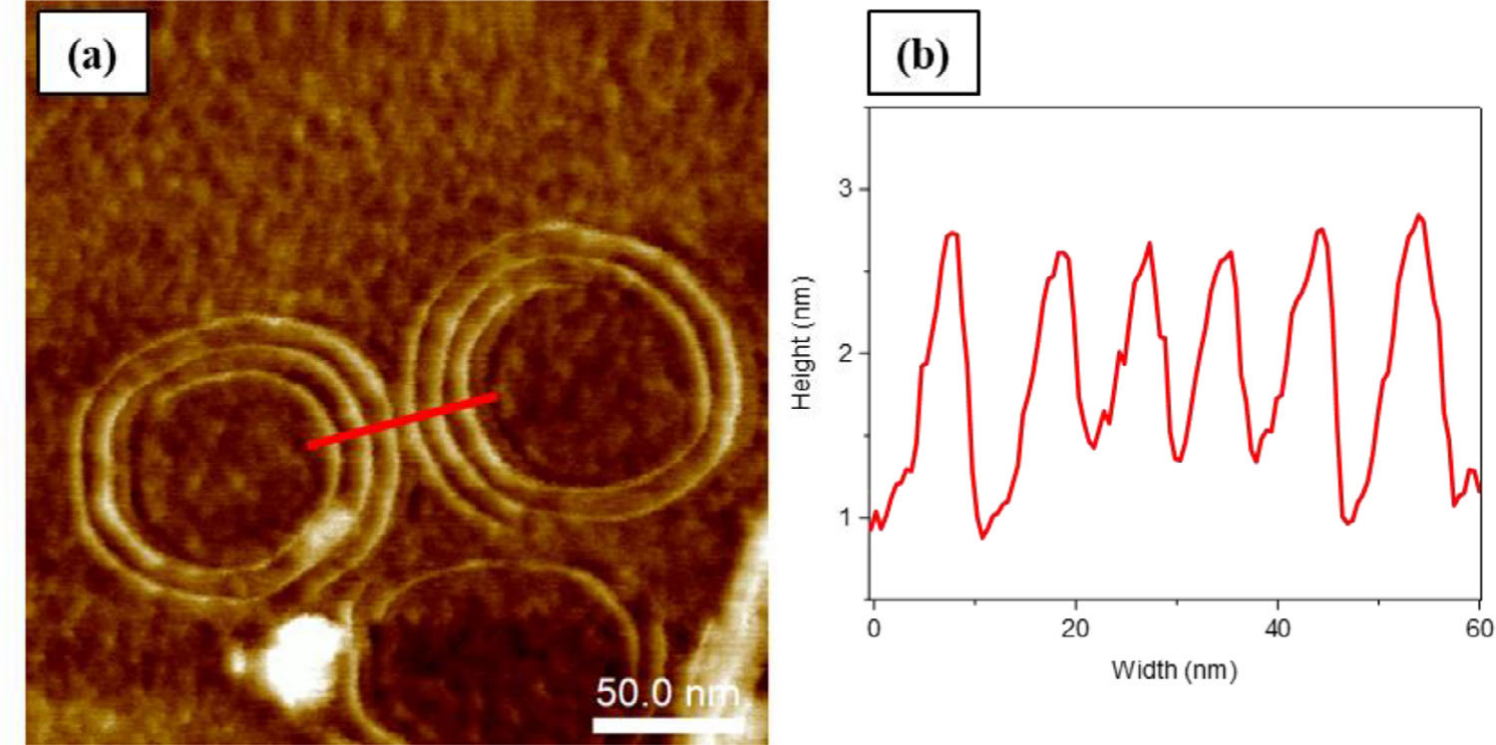
a) AFM image of the morphologies formed by the self‐assembly of **PDI‐3** in THF/H₂O (50:50) at a concentration of 5 × 10^−6^ M, using a carbon‐coated copper grid as the substrate. b) Height profile along the corresponding red line.

It is worth noting that AFM images of the PDI derivatives on a carbon‐coated copper grids were also collected. However, no well‐defined fibers were detected under these conditions for **PDI‐1** and **PDI‐2**. In contrast, fibers formed through the self‐assembly of **PDI‐3** were observable on this substrate, with **PDI‐3** forming well‐organized supramolecular fibers (coiled or spiral structures), consistent with the TEM findings, as shown in Figure 10a. The analysis of these fiber dimensions revealed that the structures exhibited lower heights (1.70 nm) and widths (6.50 nm) compared to those observed on the mica substrate, as shown in Figure 10b (red profile). This variation in dimensions is influenced by the sample preparation method and the type of substrate used. The hydrophilic nature of the mica substrate likely played a key role in promoting the formation of multiple hydrogen‐bonding interactions, thereby affecting the resulting fiber dimensions.

AFM analysis of PDI derivatives on mica substrates provided comprehensive insights into the self‐assembly behavior of **PDI‐1**, **PDI‐2**, and **PDI‐3**, supporting the structural trends observed in TEM data. Each derivative demonstrated distinct morphological characteristics, with a notable increase in curvature and structural complexity progressing from **PDI‐1** to **PDI‐3**. The transition from aligned fibers in **PDI‐1** to well‐organized coiled structures in **PDI‐3** demonstrates the marked influence of hydrophobic alkyl chains and π‐π stacking interactions on the morphology of the final self‐assembled fibers. An increase in fiber heights in **PDI‐2** and **PDI‐3** was also observed, emphasizing the role of alkyl chain length and hydrophobic interactions in promoting aggregation. Notably, **PDI‐3** (compared to **PDI‐1** and **PDI‐2**) maintained well‐defined coiled architectures across various substrates, indicating stronger interactions driving the formation of unique self‐assembled structures. These findings further highlight the influence of substituent hydrophobicity and other structural factors on the morphology and assembly behavior of PDI derivatives. It is important to note that time‐dependent AFM morphological data for **PDI‐1**, **PDI‐2**, and **PDI‐3** are provided in the Supporting Information (see Figure S29, data acquired after an approximate 6‐month interval). These results further support the exceptional stability of the morphology of the PDI assemblies over time.

Based on the analysis of AFM data and the fiber height of the assembled PDI structures, we carefully examined the curvature observed in the fibers, especially noting the significant difference in fiber structure based on differences in chemical structure. Figure S22 highlights the curvature characteristics of **PDI‐3** compared to **PDI‐1** (see Figure S22); it is noteworthy that the **PDI‐3** fibers appear thicker on the outer side of the curves (Figure S22b). This unusual morphology is clearly observed by comparing the height profiles, where a distinct curvature is evident for **PDI‐3**, as shown in Figure S22d (red profile) – and in comparison with the profile for **PDI‐1** (Figure S22b). Figure S23 provides an overview of proposed stacking modes, and the morphologies observed, providing potential explanations in terms of packing arrangements for the observed increase in curvature of the self‐assembled fibers from **PDI‐1** to **PDI‐3**. Due to the lack of observed crystallinity, these proposals are based on geometrical factors from the molecular design (please also see Table S3 for additional volumetric considerations for our molecular design).

## 3 Conclusion

In this work, we have investigated the effects of careful variation of hydrophilic and hydrophobic substituents on the properties of designed asymmetric PDIs and their resulting SMPs. The asymmetric PDIs were successfully synthesized using a one‐step approach, but did pose challenges for the purification from mixtures of products. The solution self‐assembly studies of the obtained asymmetric PDI derivatives were controlled using THF/H_2_O mixtures; it was found that specific mixtures induce the aggregation of these asymmetric PDIs at increasing ratios of water in a highly predictable fashion, as shown by repeat investigations. The hydrophobic perylene core and (systematically increased) alkyl groups promote hydrophobic and π–π interactions, while the hydrophilic OEG moieties stabilize the supramolecular polymers in the THF/H_2_O mixture through the formation of multiple hydrogen bonds. The supramolecular polymerization of the resulting SMPs, studied by temperature‐dependent UV/Vis spectroscopy, highlighted interesting routes to tuning supramolecular polymerization mechanisms: Temperature‐dependent UV/Vis absorption spectra clearly show a shift from an isodesmic polymerization process to a cooperative polymerization as the number of hydrophobic alkyl groups increases. Morphological investigation using TEM and AFM revealed the formation of different supramolecular structures, specifically with a trend to form coiled structures during their assembly process with an increase in the hydrophobicity (number and volume) of the alkyl chains. Time‐dependent AFM investigations furthermore underlined the stability of the formed structures. We envisage that this detailed study of these new asymmetric PDI systems will provide insight into the design of future SMP systems, leading to control and tunability of both SMP mechanism and the formation of intricate morphologies.

## Conflict of Interest

The authors declare no conflict of interest.

## Supporting information



Supporting Information

## Data Availability

The data that support the findings of this study are available in the supplementary material of this article.
