# Supplementary material for: Self‐Assembly, Aggregation Mechanisms, and Morphological Properties of Asymmetric Perylene Diimide‐based Supramolecular Polymers
Source: Chemistry. 2025 Jul 2;31(41):e202501317. doi: 10.1002/chem.202501317 (PMC12284623; doi:10.1002/chem.202501317)
Supplement: Supplementary file 1 — Supporting Information [file CHEM-31-e202501317-s001.pdf]

Supporting Information

Self-Assembly, Aggregation Mechanisms, and Morphological Properties of Asymmetric Perylene Diimide-based Supramolecular Polymers

Helal S. Alharbi,[a,b] Xue Fang,[a] Robert L. Harniman,[a] and Charl F. J. Faul[a]*

[a] School of Chemistry, University of Bristol, Bristol, BS8 1TS, UK

[b] Department of Chemistry, College of Science, Qassim University, Buraydah 52571, Saudi Arabia

Email: charl.faul@bristol.ac.uk

Table of contents

1 Materials and methods 3

1.1 Materials 3

1.2 Nuclear magnetic resonance spectroscopy 3

1.3 Mass spectrometry 3

1.4 Ultraviolet-visible spectroscopy 3

1.5 Transmission electron microscopy 3

1.6 Atomic force microscopy 4

2 Synthesis 5

2.1 Synthesis of amines 5

2.1.1 Synthesis of methyl 4-(dodecyloxy) benzoate, (Alkoxy-2) 5

2.1.2 Synthesis of alkoxy amine, (Amine-2) 5

2.1.3 Synthesis of methyl 3,5-bis(dodecyloxy)benzoate, (Alkoxy-3) 6

2.1.4 Synthesis of dialkoxy amine, (Amine-3) 6

2.2 Synthesis of asymmetric PDIs 7

2.2.1 Synthesis of PDI-1 7

2.2.2 Synthesis of PDI-2 8

2.2.3 Synthesis of PDI-3 8
3 Characterization results 10
4 References 31

1 Materials and methods

1.1 Materials

All reactions were carried out under nitrogen gas protection. All chemicals were used as stated by the manufacturer unless advised otherwise, and all solvents were analytical grade quality without additional purification.

1.2 Nuclear magnetic resonance spectroscopy

Nuclear magnetic resonance (NMR) spectra were measured using a 400 MHz Jeol ECZ400 or a 500 MHz Bruker 500 spectrometer, and they were analyzed by MestReNova software. Resonances of the samples were referenced to the residual solvent. The limited availability of products, along with their high molecular weights, contributes to deviations in certain integrals. These deviations are especially evident in the alkyl proton peaks.

1.3 Mass spectrometry

Mass spectrometry was performed either on matrix-assisted laser desorption/ionization time-of-flight mass spectrometry (MALDI-ToF) or electrospray ionization (ESI). A solution of *trans*-2-[3-(4-*tert*-Butylphenyl)-2-methyl-2-propenylidene]malononitrile (DCTB) in chloroform or dichloromethane was used as matrix for all sample on MALDI. Using a ratio of 1:1, the matrix and sample solutions (0.1 mg mL^{-1}) were combined, and roughly 2 μl of this mixture was then applied to a polished stainless steel plate and left to dry. When the ESI was used, the sample solution (0.1 mg mL^{-1}) was prepared in methanol/dichloromethane at a ratio of 1:1.

1.4 Ultraviolet-visible spectroscopy

The ultraviolet-visible (UV/Vis) spectroscopy measurements were performed using a Shimadzu UV-2600 spectrometer equipped with an ISR-2600 integrating sphere attachment. A quartz cuvette with a 10 mm path length was utilized for all measurements. Variable temperature UV/Vis measurements were performed using a PerkinElmer Lambda 35 spectrophotometer connected to a Peltier temperature controller. Spectra were collected at 5 K intervals, waiting 5 min for thermal equilibration between each measurement.

1.5 Transmission electron microscopy

Transmission electron microscopy (TEM) was carried out using a JEOL JEM-2100F operating at a voltage of 200 kV and equipped with a Gatan Orius SC1000 camera. Samples were prepared by

drop casting 5–10 μL of solution on a carbon-coated copper grid, placed on a filter paper to facilitate solvent removal. ImageJ software was used to analyze TEM images. Length and width measurements were obtained by tracing > 100 structures by hand.

To prepare carbon-coated TEM grids, a Q150TES instrument from Quorum Technologies Ltd. was used to coat high-purity graphite onto mica. Films were then floated on deionised water and placed onto either 400 or 600 mesh copper grids from Agar Scientific.

1.6 Atomic force microscopy

Atomic force microscopy (AFM) was conducted in ambient environment using a Multi-Mode VIII microscope with a Nanoscope V controller under PeakForce feedback control. A Fastscan head unit was utilized in conjunction with SCANASYST-AIR-HR cantilevers with nominal spring constant and tip radius of 0.4 N m^{-1} and 2 nm respectively. 5–10 μL of sample solution was drop-cast onto mica substrate, allowing it to dry naturally.

2 Synthesis

2.1 Synthesis of amines

Amine substituents were prepared and purified in our lab according to standard reported procedures.[1,2] Jarrett-Wilkins and Symons publications provide detailed procedures for the synthesis of **Amine-1** and **Amine-4**.[1,2]

2.1.1 Synthesis of methyl 4-(dodecyloxy) benzoate, (Alkoxy-2)

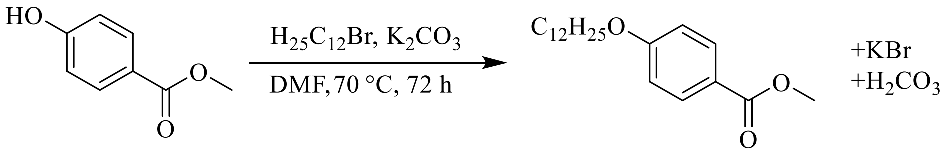

Methyl 4-hydroxybenzoate (5.0 g, 32.8 mmol, 1.0 eq.) and K_2CO_3 (11.3 g, 82 mmol, 2.5 eq.) were dried in vacuo and then dried dimethylformamide (DMF) (50 mL) was added. 1-bromododecane (16.3 g, 65.6 mmol, 2.0 eq.) was added slowly over 10 min and the mixture was then heated up to 70 °C for 3 days, then cooled to room temperature. Water (200 mL) was added to the reaction mixture. The mixture was stirred for 30 min at room temperature. The white precipitate was collected by vacuum filtration and washed with water (200 mL). The residue was recrystallized twice from acetone to yield the product. The yield was ca. 90%.

^{1}H NMR (400 MHz, $CDCl_3$) δ (ppm) = 7.96 (d, J = 8.9 Hz, 2H), 6.91 (d, J = 8.9 Hz, 2H), 4.00 (t, J = 6.6 Hz, 3H), 3.88 (s, 3H), 1.79 (m, 2H), 1.45 (m, 2H), 1.26 (m, 16H), 0.88 (t, J = 6.8, 3H).

ESI calculated for $C_{20}H_{32}O_3$: 321.24 *m/z* $[M + H]^+$, found 321.24 *m/z* $[M + H]^+$.

2.1.2 Synthesis of alkoxy amine, (Amine-2)

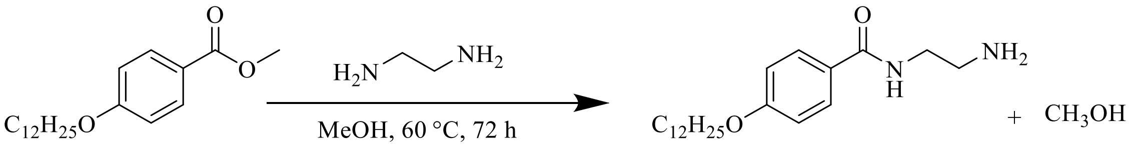

Alkoxy-2 **Amine-2**

Methyl 4-(dodecyloxy) benzoate, **Alkoxy-2**, (2.0 g, 6.24 mmol, 1.0 eq.) dissolved in dry MeOH (30 mL) under nitrogen atmosphere, and ethylene diamine (31.24. mL, 468 mmol, 75 eq.) were added to a round bottom flask attached with a reflux condenser. The reaction mixture was stirred and refluxed at 60 °C for three days. After cooling to room temperature, distilled water (100 mL) was added to the mixture and the crude product was extracted with

dichloromethane (DCM) (300 mL). The organic phase was washed with brine and water. The solvent of the combined phases was removed in vacuo, and the reaction mixture was dried. The crude product was purified using column chromatography (70:20:10, DCM: Hexane: MeOH) to provide the purified product. The yield was ca. 60%.

^{1}H NMR (400 MHz, $CDCl_3$) δ (ppm) = 7.75 (d, J = 8.8 Hz, 2H), 6.92 (d, J = 8.8 Hz, 2H), 3.98 (t, J = 6.6 Hz, 2H), 3.49 (q, J = 5.9 Hz, 2H), 2.94 (t, J = 5.9 Hz, 2H), 1.77 (m, 2H), 1.45 (m, 2H), 1.26 (m, 16H), 0.88 (t, J = 7.00, 3H).

ESI calculated for $C_{21}H_{36}N_2O_3$: 349.29 *m/z* $[M + H]^+$, found 349.28 *m/z* $[M + H]^+$.

2.1.3 Synthesis of methyl 3,5-bis(dodecyloxy)benzoate, (Alkoxy-3)

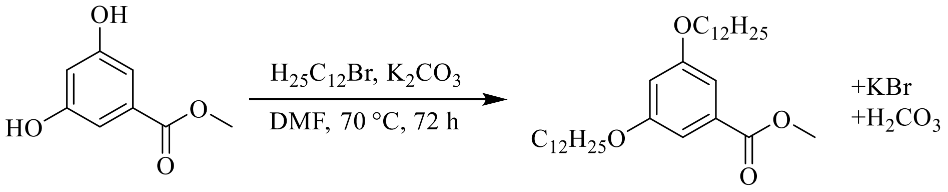

Methyl 3,5-dihydroxybenzoate (5.0 g, 29.7 mmol, 1.0 eq.) and K_2CO_3 (10.27 g, 74.33 mmol, 2.5 eq.) were dried in vacuo and then dried DMF (50 mL) was added. 1-bromododecane (18.5 g, 74.33 mmol, 2.5 eq.) was added slowly over 10 min and the mixture was then heated up to 70 °C for 3 days, then cooled to room temperature. Water (200 mL) was added to the reaction mixture. The mixture was stirred for 30 min at room temperature, with the white precipitate collected by vacuum filtration and washed with water (200 mL). The residue was recrystallized twice from acetone to yield the product. The yield was 80%.

^{1}H NMR (400 MHz, $CDCl_3$) δ (ppm) = 7.14 (d, J = 2.4 Hz, 2H), 6.62 (t, J = 2.4 Hz, 1H), 3.95 (t, J = 6.5 Hz, 4H), 3.88 (s, 3H), 1.76 (m, 4H), 1.43 (m, 4H), 1.25 (m, 32H), 0.87 (t, J = 7.0, 6H).

ESI calculated for $C_{32}H_{56}O_4$: 505.43 *m/z* $[M + H]^+$, found 505.42 *m/z* $[M + H]^+$.

2.1.4 Synthesis of dialkoxy amine, (Amine-3)

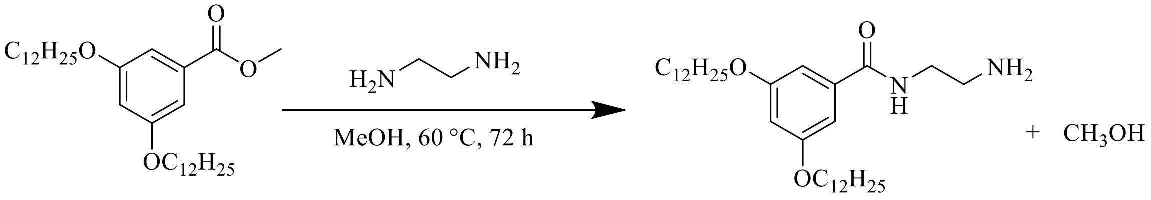

Alkoxy-3 **Amine-3**

Benzoate ester, **Alkoxy-3**, (0.5 g, 1.0 mmol, 1.0 eq.) was dissolved in dry MeOH (15 mL) under nitrogen atmosphere, and ethylene diamine (5.00 mL, 75 mmol, 75 eq.) was added to a round bottom flask attached with a reflux condenser. The reaction mixture was stirred and refluxed at 60 °C for three days. After cooling to room temperature, distilled water (100 mL) was added to the mixture and the crude product was extracted with DCM (300 mL) . The organic phase was washed with brine and water, the solvent of the combined phases removed in vacuo, and the reaction mixture was dried. The crude product was purified using column chromatography (90:10, DCM: MeOH) to provide the purified product. The yield was ca. 60%.

^{1}H NMR (400 MHz, $CDCl_3$) δ (ppm) = 6.88 (d, J = 2.2 Hz, 2H), 6.55 (t, J = 2.3 Hz, 1H), 3.96 (t, J = 6.6 Hz, 4H), 3.48 (q, J = 5.8 Hz, 2H), 2.93 (t, J = 5.8 Hz, 2H), 1.76 (m, 4H), 1.43 (m, 4H), 1.26 (m, 32H), 0.88 (t, J = 7.0, 6H).

ESI calculated for $C_{33}H_{60}N_2O_3$: 533.47 *m/z* $[M + H]^+$, found 533.46 *m/z* $[M + H]^+$.

2.2 Synthesis of asymmetric PDIs

2.2.1 Synthesis of PDI-1

Perylene-3,4,9,10-tetracarboxylic dianhydride (255 mg, 0.65 mmol, 1 eq.), and 4-alkoxy amine, **Amine-2,** (250 mg, 0.71 mmol, 1.1 eq.) were dried in a flask under vacuum, then placed under nitrogen atmosphere for 5 min. Benzyl amide, **Amine-1**, (604 mg, 0.65 mmol, 1 eq.), zinc acetate (1.30 mmol, 2.3 eq., 248 mg) and 1-methylimidazole (3.62 mL, 35 mmol, 70 eq.) were added to a round bottom flask fitted with a reflux condenser. The reaction mixture was heated and stirred for 24 h at 130 °C and then cooled to room temperature. DCM (25 mL) was added, and the mixture was briefly sonicated. The mixture was diluted with DCM (100 mL) and washed with aqueous HCl (2M, 100 mL), using a small amount of isopropyl alcohol to break an emulsion if necessary. The aqueous phase was extracted with DCM (2 × 10 mL) and the combined organic phases were then dried over anhydrous $MgSO_4$, filtered, and the solvent was removed. The crude product was purified by silica gel column chromatography (DCM:MeOH:Et_3N = 90:9:1), followed by preparative TLC ($CHCl_3$) to give the pure product. The yield was 7%.

^{1}H NMR (400 MHz, $CDCl_3$) δ (ppm) = 8.72–8.61 (m, 8H, perylene-H), 7.72 (d, J = 8.5 Hz, 2H, phenyl-H), 7.05 (d, J = 11.2 Hz, 2H, phenyl-H), 6.89 (d, J = 9.0 Hz, 2H, phenyl-H), 6.57 (s, 1H, phenyl-H), 4.57 (m, 4H, N-CH_2), 4.12 (m, 4H, OEG-H), 3.96(m, 2H, -CH_2), 3.85 (m, 4H, OEG-

H), 3.75–3.51 (m, 40H, OEG-H, N-CH_2), 3.36 (m, 6H, OEG-O-CH_3), 1.85 – 1.75 (m, 2H, -CH_2), 1.44 (m, 2H, -CH_2),1.26 (m, 34H, -CH_2), 0.88 (m, 3H, -CH_3).

MALDI-ToF MS calculated for $C_{86}H_{116}N_4O_{24}$: 1612.8 *m/z* $[M + Na]^+$, found 1612.0 $[M + Na]^+$, mass corresponds to OEG DP_n of 7 and 8. Calculated for $C_{88}H_{120}N_3O_{25}$: 1656.9 *m/z* $[M + Na]^+$, found 1656.0 $[M + Na]^+$, mass corresponds to OEG DP_n of 8 and 8.

2.2.2 Synthesis of PDI-2

Perylene-3,4,9,10-tetracarboxylic dianhydride (193 mg, 0.49 mmol, 1 eq.), and 3,5-dialkoxy amine, **Amine-3**, (290 mg, 0.54 mmol, 1.1 eq.) were dried in a flask under vacuum, then placed under nitrogen atmosphere for 5 min. Benzyl amide, **Amine-1**, (457 mg, 0.49 mmol, 1 eq.), zinc acetate (215 mg, 2.3 eq., 1.13 mmol) and 1-methylimidazole (3.5 mL, 34.4 mmol, 70 eq.) were added to a round bottom flask fitted with a reflux condenser. The reaction mixture was heated and stirred for 24 h at 130 °C and then cooled to room temperature. DCM (25 mL) was added, and the mixture briefly sonicated. The mixture was diluted with DCM (100 mL) and washed with aqueous HCl (2M, 100 mL), using a small amount of isopropyl alcohol to break an emulsion if necessary. The aqueous phase was extracted with DCM (2 × 10 mL) and the combined organic phases were then dried over anhydrous $MgSO_4$, filtered, and the solvent removed. The crude product was purified by silica gel column chromatography (DCM:MeOH:Et_3N = 90:9:1), followed by preparative TLC ($CHCl_3$) to give the pure product. The yield was 16%.

^{1}H NMR (400 MHz, $CDCl_3$) δ (ppm) = 8.72–8.58 (m, 8H, perylene-H), 6.88 (m, 4H, phenyl-H), 6.57 (m, 2H, phenyl-H), 4.55 (m, 4H, N-CH_2), 4.12 (m, 4H, OEG-H), 4.00 – 3.93 (m, 4H, -CH_2), 3.84 (m, 4H, OEG-H), 3.74–3.48 (m, 40H, OEG-H, N-CH_2), 3.36 (m, 6H, OEG-O-CH_3), 1.80 – 1.73 (m, 4H, -CH_2), 1.44 (m, 4H, -CH_2),1.26 (m, 34H, -CH_2), 0.88 (m, 6H, -CH_3).

MALDI-ToF MS calculated for $C_{98}H_{140}N_4O_{25}$: 1795.9 *m/z* $[M + Na]^+$, found 1796.1 $[M + Na]^+$, mass corresponds to OEG DP_n of 7 and 8. Calculated for $C_{88}H_{120}N_3O_{25}$: 1840.0 *m/z* $[M + Na]^+$, found 1840.1 $[M + Na]^+$, mass corresponds to OEG DP_n of 8 and 8.

2.2.3 Synthesis of PDI-3

Perylene-3,4,9,10-tetracarboxylic dianhydride (100 mg, 0.25 mmol, 1 eq.), and trialkoxy amine, **Amine-4**, (200 mg, 0.28 mmol, 1.1 eq.) were dried in a flask under vacuum, then placed under nitrogen atmosphere for 5 min. Benzyl amide, **Amine-1** (248 mg, 0.28 mmol, 1.1 eq.), and 1-methylimidazole (2.79 mL, 35 mmol, 70 eq.) were added to a round bottom flask fitted with a

reflux condenser. The reaction mixture was heated and stirred for 2 h at 130 °C and then cooled to room temperature. DCM (25 mL) was added, and the mixture briefly sonicated. The mixture was diluted with DCM (100 mL) and washed with aqueous HCl (2M, 100 mL), using a small amount of isopropyl alcohol to break an emulsion if necessary. The aqueous phase was extracted with DCM (2 × 10 mL) and the combined organic phases were then dried over anhydrous $MgSO_4$, filtered, and the solvent removed. The crude product was purified by silica gel column chromatography (DCM:MeOH:Et_3N = 90:9:1), followed by preparative TLC ($CHCl_3$) to give the pure product. The yield was 15%.

^{1}H NMR (400 MHz, $CDCl_3$) δ (ppm) = 8.63–8.50 (m, 8H, perylene-H), 6.99 (s, 2H, phenyl-H), 6.91 (s, 2H, phenyl-H), 6.58 (s, 1H, phenyl-H), 4.54 (m, 4H, N-CH_2), 4.11 (m, 4H, OEG-H), 4.00 – 3.94(m, 6H, -CH_2), 3.88 (m, 4H, OEG-H), 3.83 (m, 4H, OEG-H), 3.72–3.60 (m, 40H, OEG-H, N-CH_2), 3.36 (m, 6H, OEG-O-CH_3), 1.83 – 1.69 (m, 6H, -CH_2), 1.46 (m, 6H, -CH_2),1.25 (m, 54H), 0.86 (m, 9H, -CH_3).

MALDI-ToF MS calculated for $C_{110}H_{164}N_4O_{26}$: 1980.15 *m/z* $[M + Na]^+$, found 1980.79 $[M + Na]^+$, mass corresponds to OEG DP_n of 7 and 8. Calculated for $C_{112}H_{168}N_4O_{27}$: 1400.5 *m/z* $[M + Na]^+$, found 2024.82 $[M + Na]^+$, mass corresponds to OEG DP_n of 8 and 8.

3 Characterization results

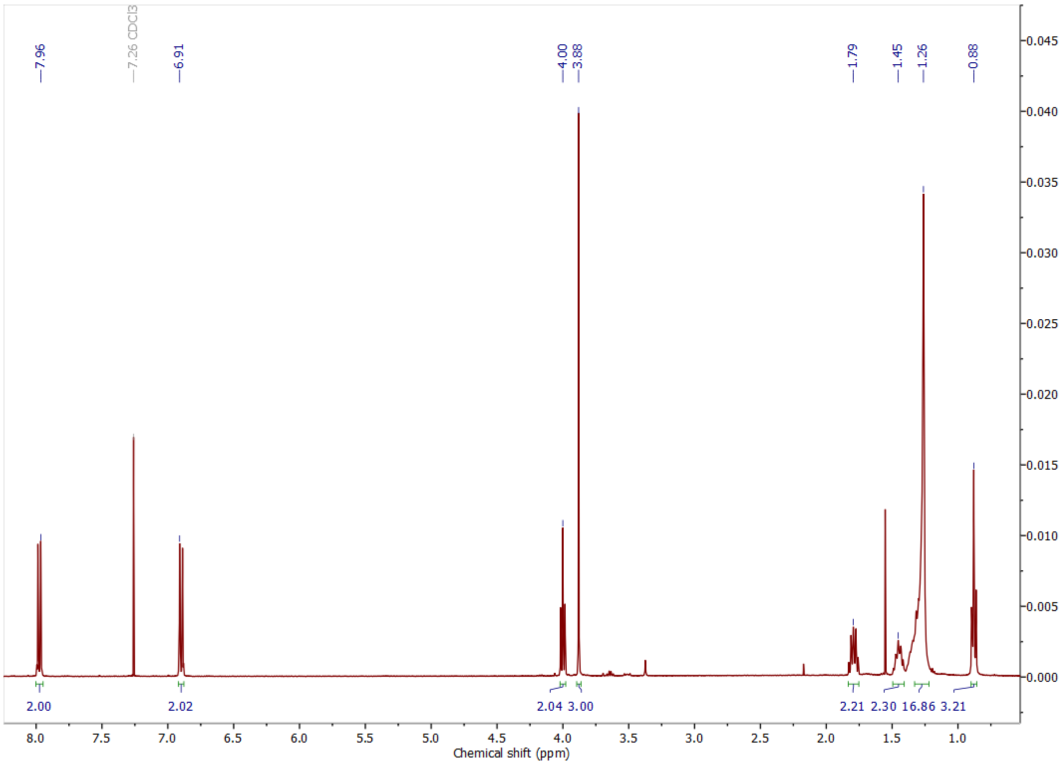

Figure S1: ^{1}H NMR spectrum of **Alkoxy-2**.

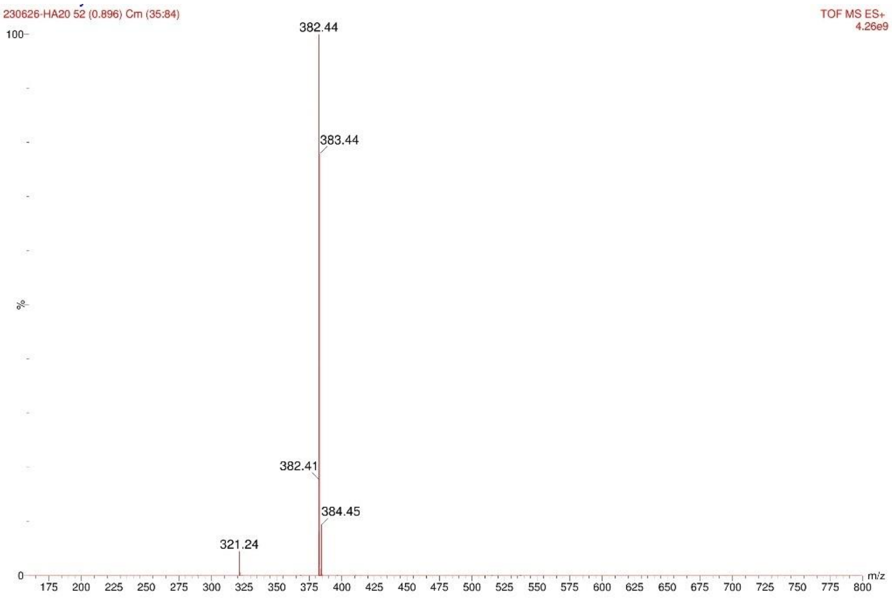

Figure S2: ESI mass spectrum of **Alkoxy-2**.

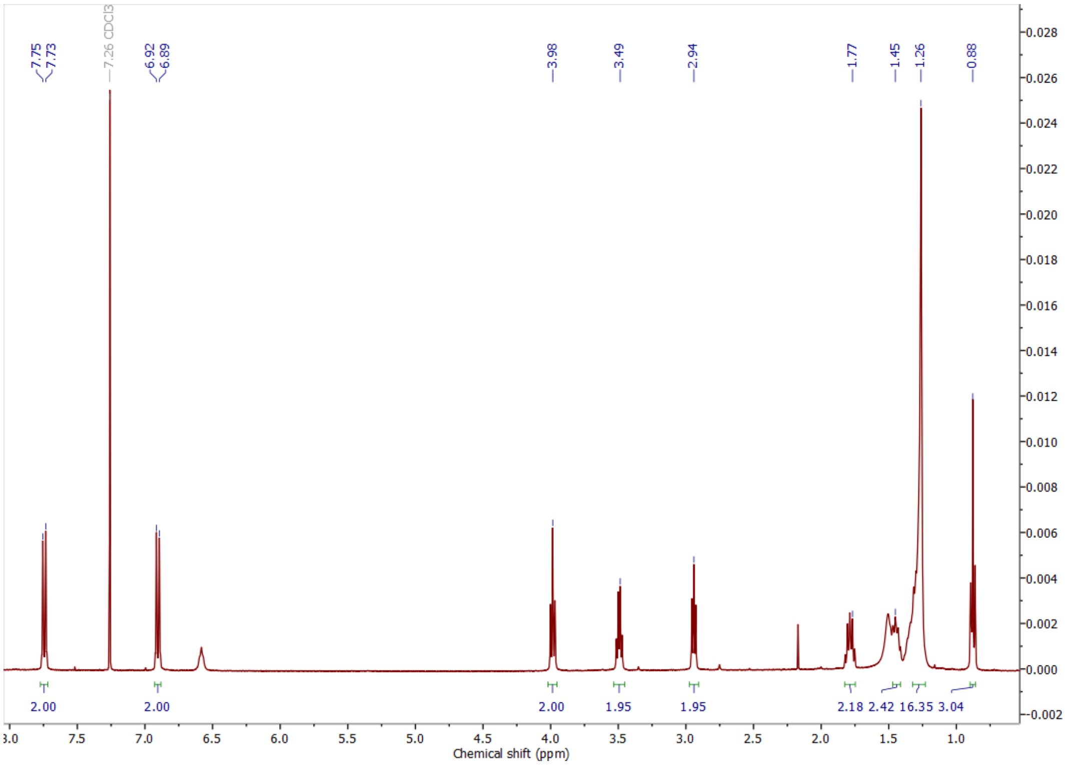

Figure S3: ^{1}H NMR spectrum of **Amine-2**.

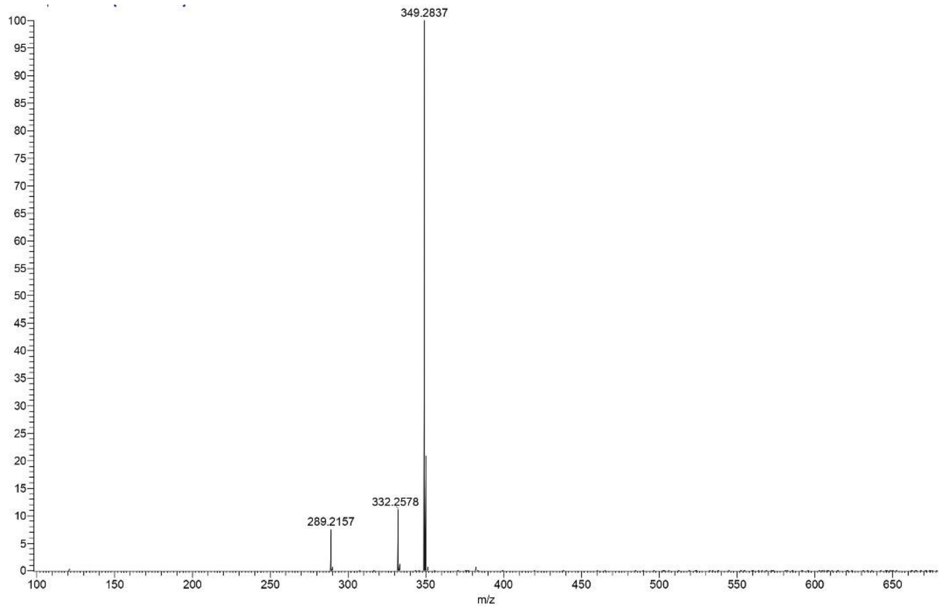

Figure S4: ESI mass spectrum of **Amine-2**.

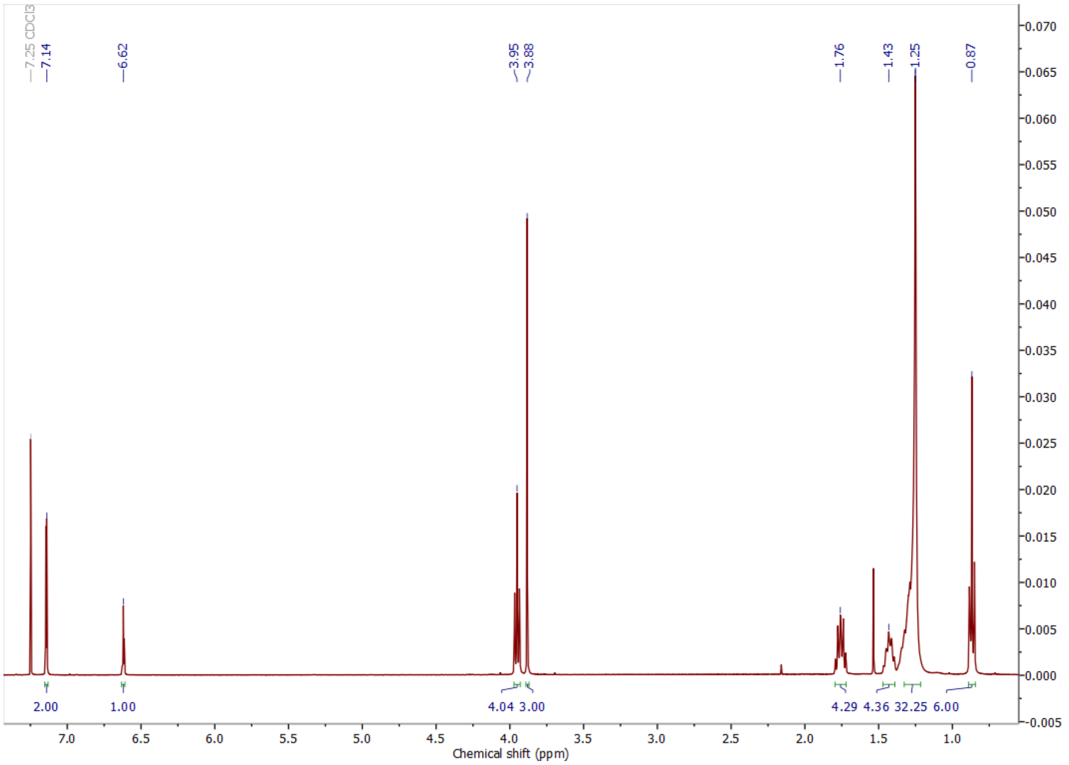

Figure S5: ^{1}H NMR spectrum of **Alkoxy-3**.

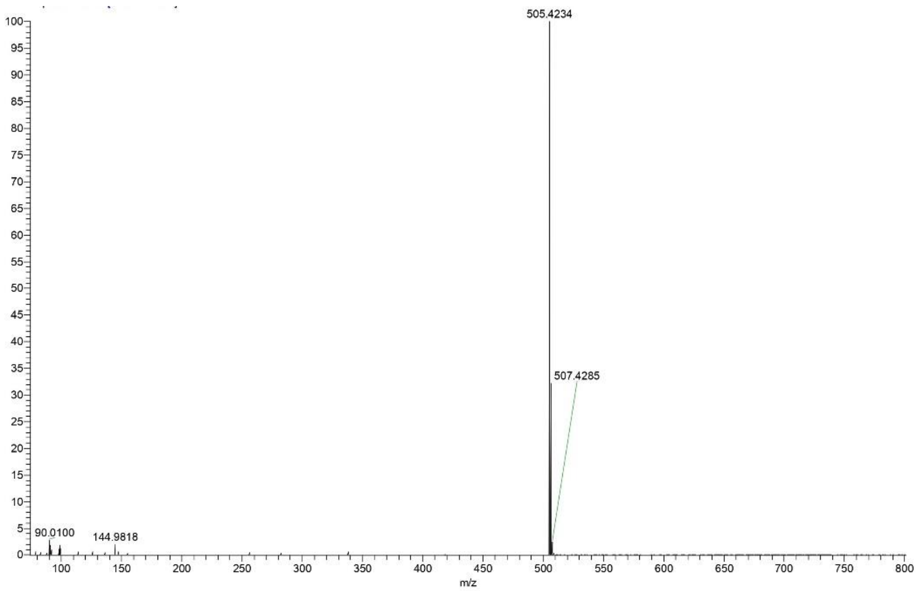

Figure S6: ESI mass spectrum of **Alkoxy-3**.

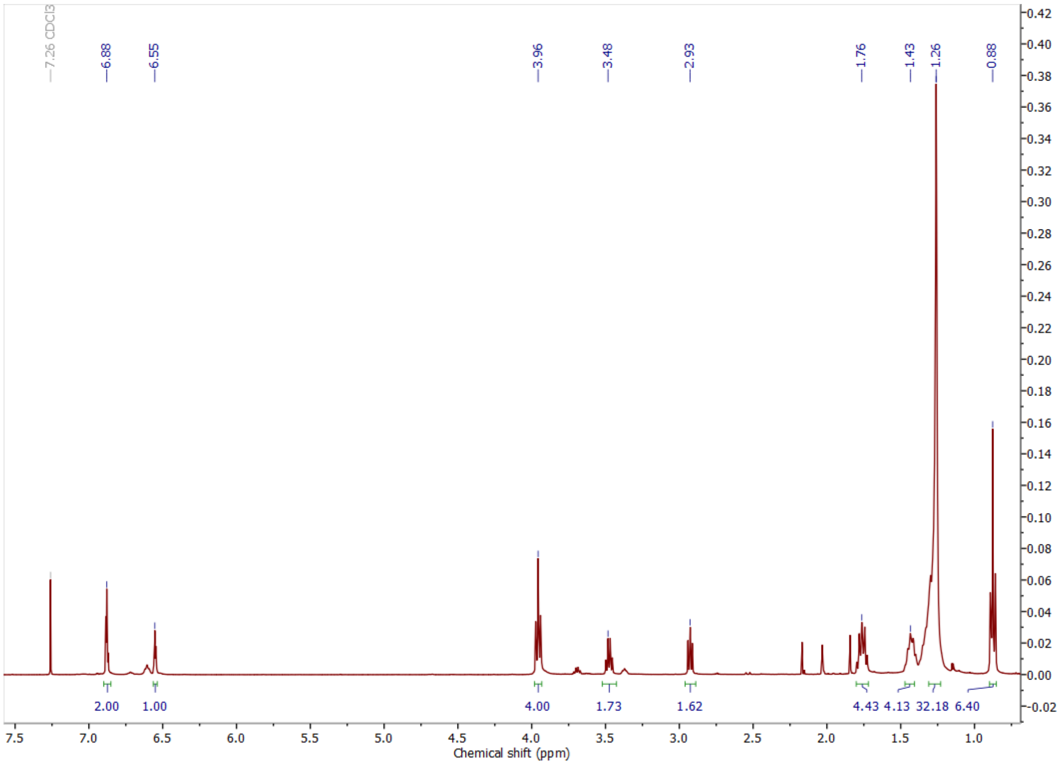

Figure S7: ^{1}H NMR spectrum of **Amine-3**.

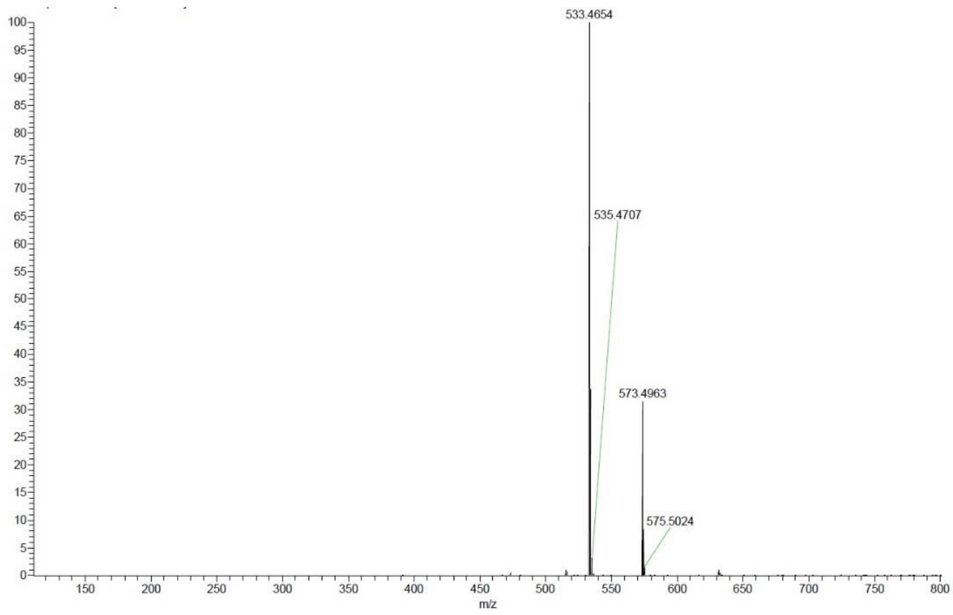

Figure S8: ESI mass spectrum of **Amine-3**.

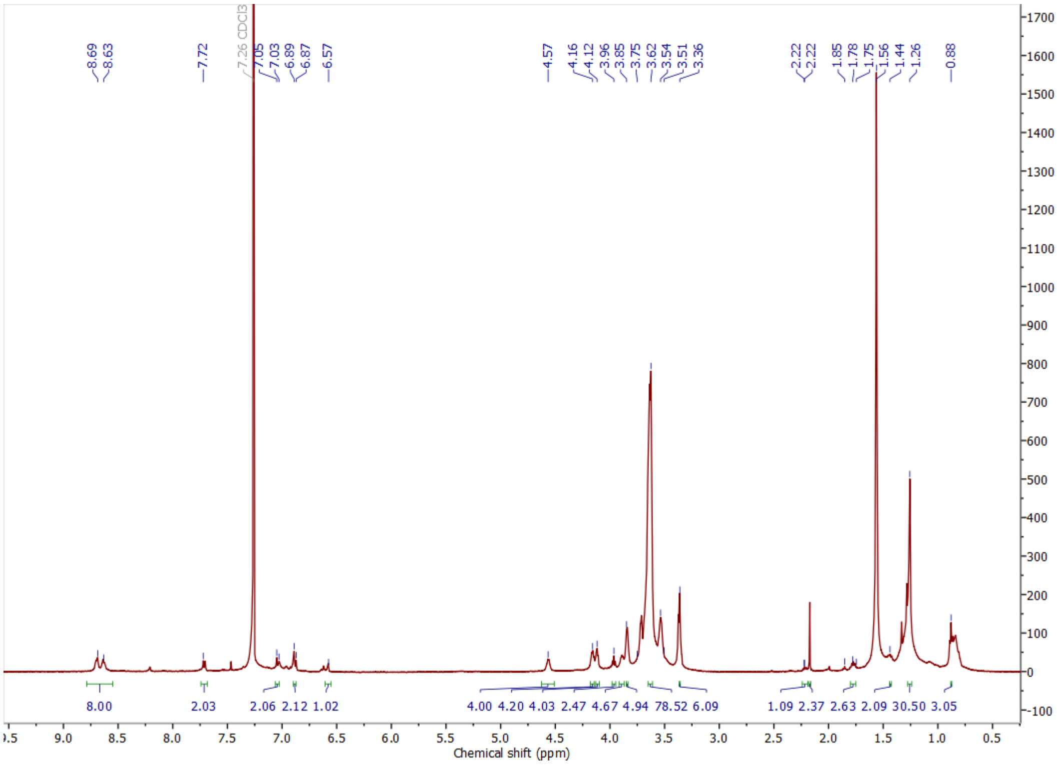

Figure S9: ^{1}H NMR spectrum of **PDI-1**.

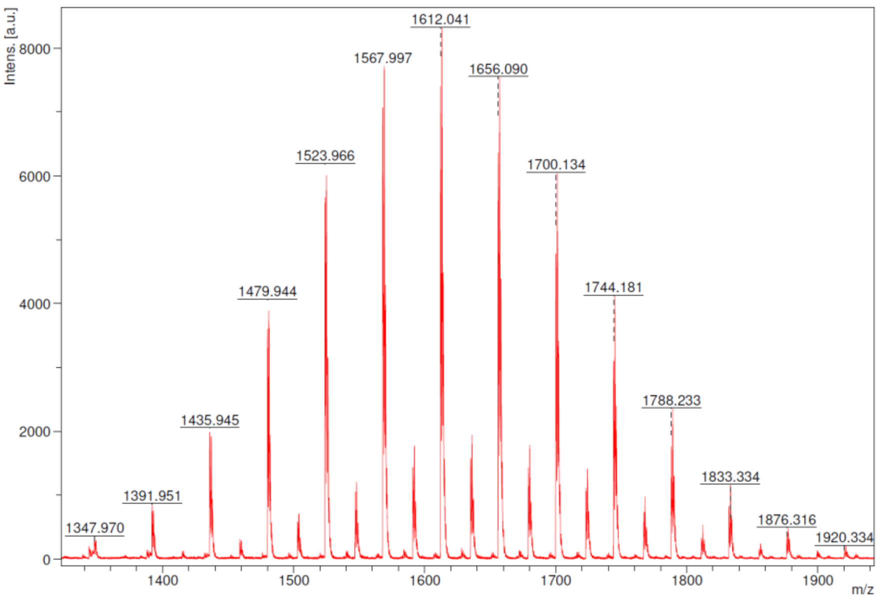

Figure S10: MALDI-ToF mass spectrum of **PDI-1**.

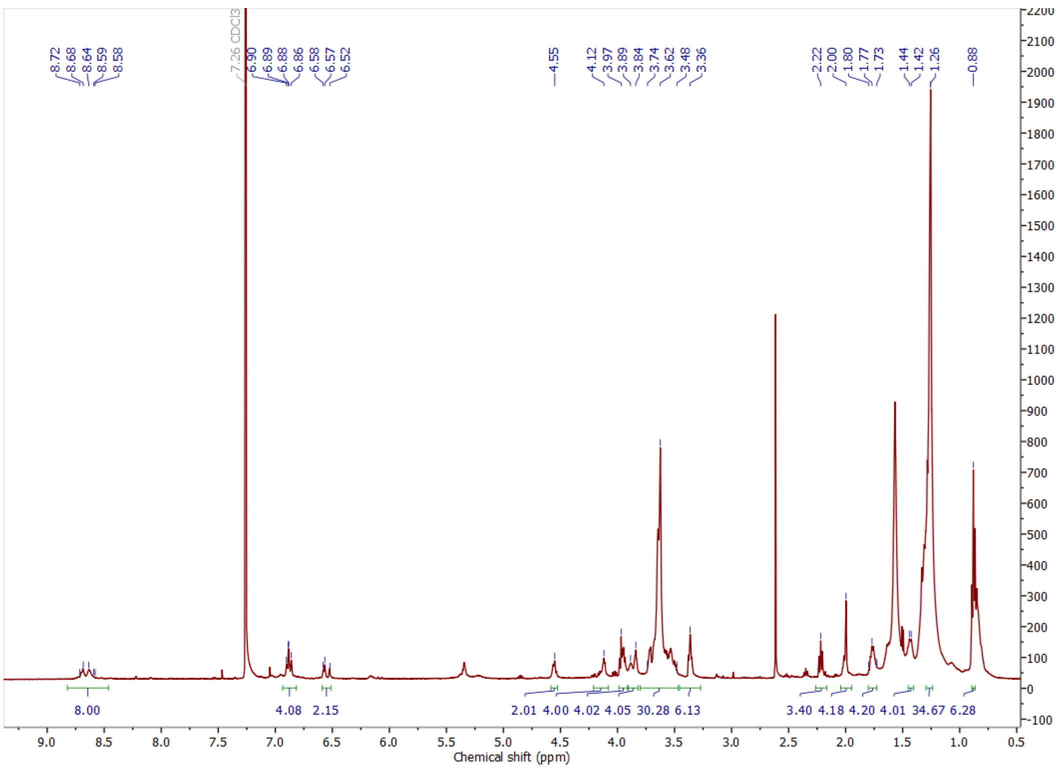

Figure S11: ^{1}H NMR spectrum of PDI-2.

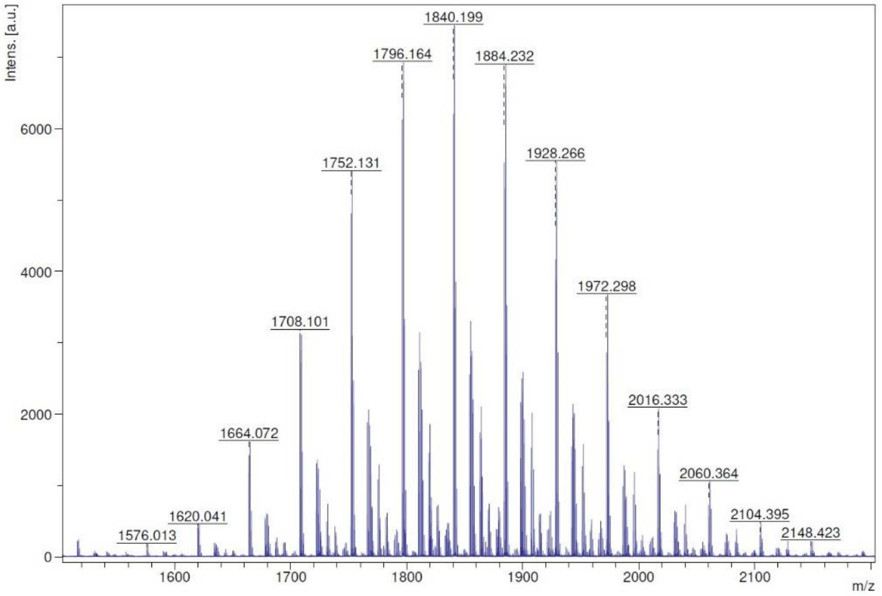

Figure S12: MALDI-ToF mass spectrum of **PDI-1**.

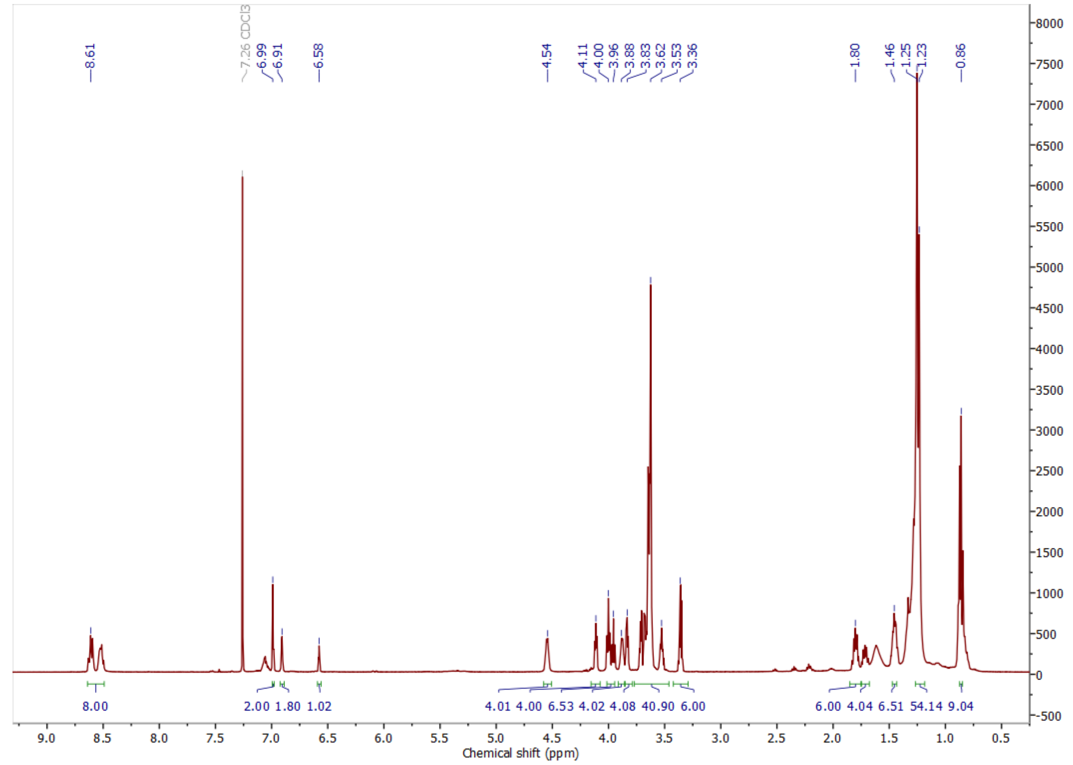

Figure S13: ^{1}H NMR spectrum of **PDI-3**.

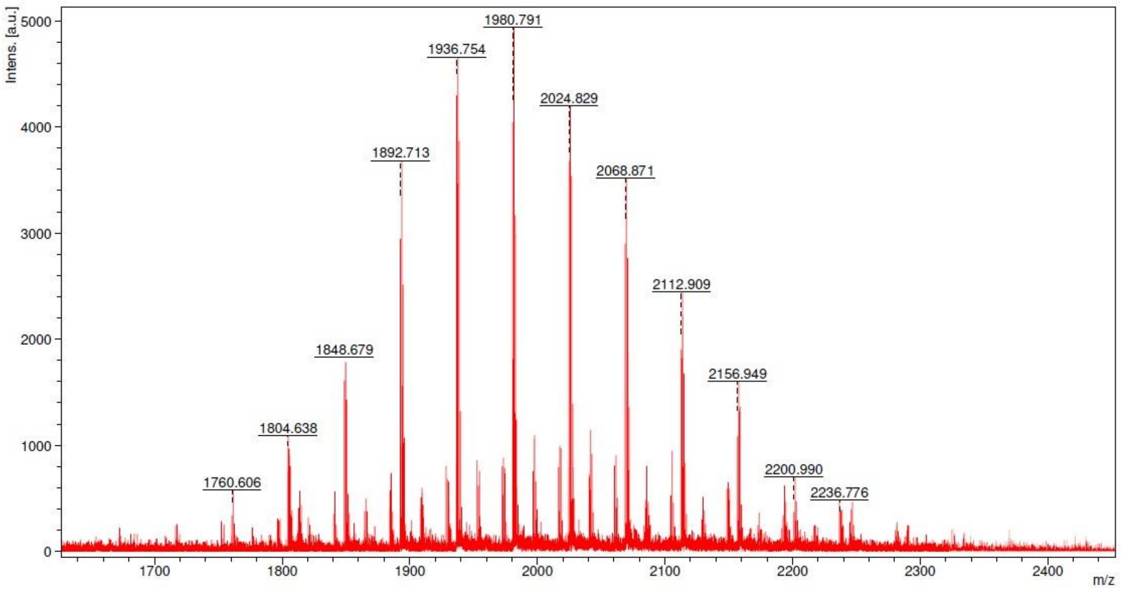

Figure S14: MALDI-ToF mass spectrum of **PDI-1**.

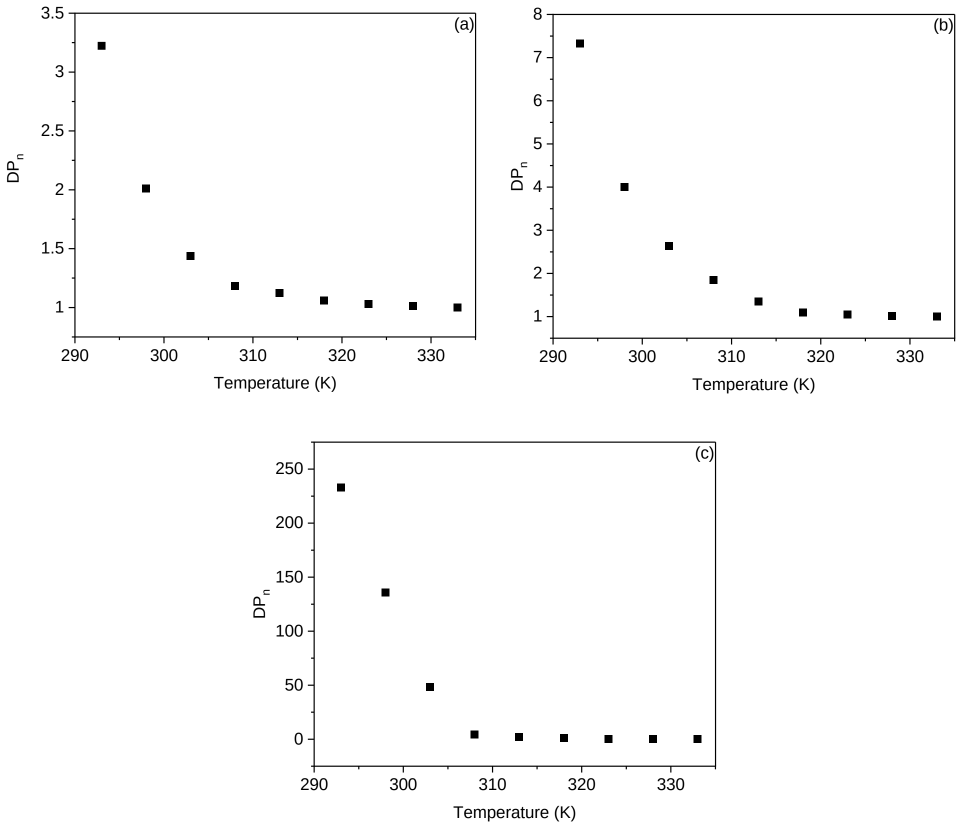

Figure S15. Plots of degree of polymerization (DP_n) as a function of temperature of (a) **PDI-1**, (b) **PDI-2**, and (c) **PDI-3** upon heating from 293 K to 333 K.

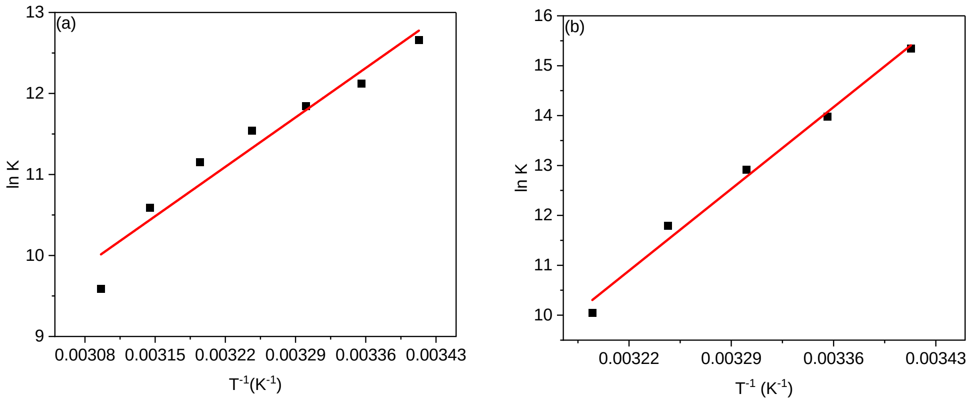

Figure S16. Van't Hoff plots for (a) **PDI-1**, and (b) **PDI-2**. All data obtained from temperature-dependent UV/Vis spectroscopy measurements.

Table S1. Summary of the dimensions (length and width) of **PDI-1**, **PDI-2**, and **PDI-3** compounds obtained from TEM images. Additionally, the table provides the observed morphology type for each compound.

Sample	Dimensions	Mean	Minimum	Maximum	Morphology
**PDI-1**	Length (µm)	2.00	0.27	5.92	Ribbon-like
	Width (nm)	40	8.00	105	
**PDI-2**	Length (µm)	1.34	0.50	2.74	Partially coiled
	Width (nm)	31	17	46	
**PDI-3**	Length (µm)	0.64	0.16	1.54	Coiled and spiral
	Width (nm)	11	6.00	16	

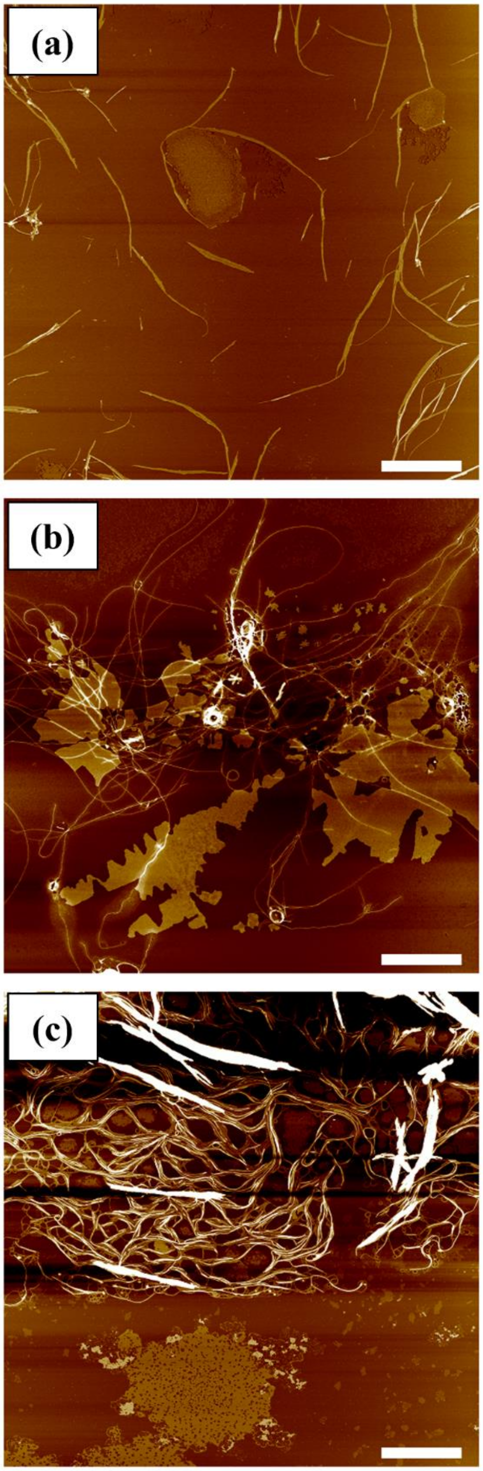

Figure S17. AFM images of the morphologies formed by the self-assembly of (a) **PDI-1** in THF/H_2O (30:70), (b) **PDI-2** in THF/H_2O (40:60), and (c) **PDI-3** in THF/H_2O (50:50) at a

concentration of 5 $\times 10^{-6}$ M. Scale bars in a, b, and c = 2 μm. The *z*-range is 10 nm for (a), 16 nm for (b), and 10 nm for (c).

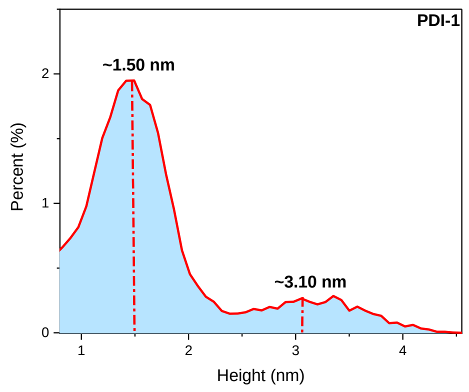

Figure S18. Height distribution histogram of **PDI-1** obtained from a typical AFM image, where the first peak indicates the typical fiber heights (~1.50 nm) of the morphologies formed through the self-assembly of **PDI-1** in a THF/H_2O (30:70) mixture.

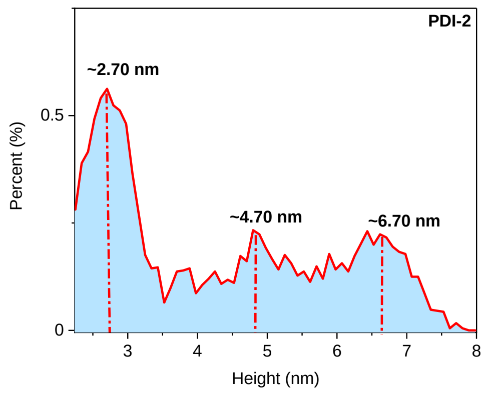

Figure S19. Height distribution histogram of **PDI-2** obtained from a typical AFM image, where the first peak indicates the typical fiber heights (~2.70 nm) of the morphologies formed through the self-assembly of **PDI-3** in a THF/H_2O (40:60) mixture.

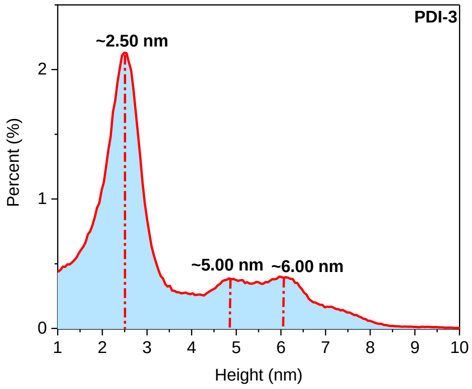

Figure S20. Height distribution histogram of **PDI-3** obtained from a typical AFM image, where the first peak indicates the typical fiber heights (~2.50 nm) of the morphologies formed through the self-assembly of **PDI-3** in a THF/H_2O (50:50) mixture.

Table S2. Summary of the dimensions (length and width) of **PDI-1**, **PDI-2**, and **PDI-3** compounds obtained from AFM images. Additionally, the table provides the observed morphology type for each compound.

Sample	Dimensions	Typical fiber	Extending to	Morphology
**PDI-1**	Height (nm)	1.50	3.5	Aligned
	Width (nm)	13	75	
**PDI-2**	Height (nm)	2.70	7.80	Wavy and curved
	Width (nm)	13	65	
**PDI-3**	Height (nm)	2.5	9.50	Coiled
	Width (nm)	20	80	

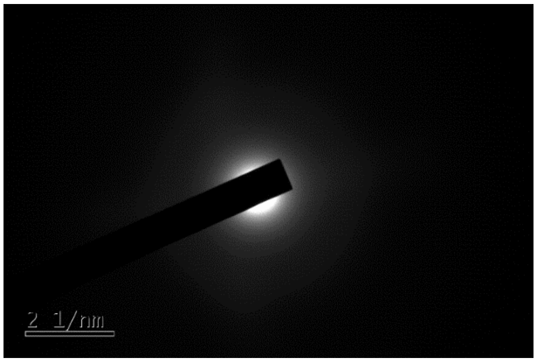

Figure S21. A typical SAED frame recorded for nanofibers formed by **PDI-3** in THF/H_2O (50:50) at a concentration of 5 × 10^{-6} M, indicating the absence of clear diffraction patterns in the data.

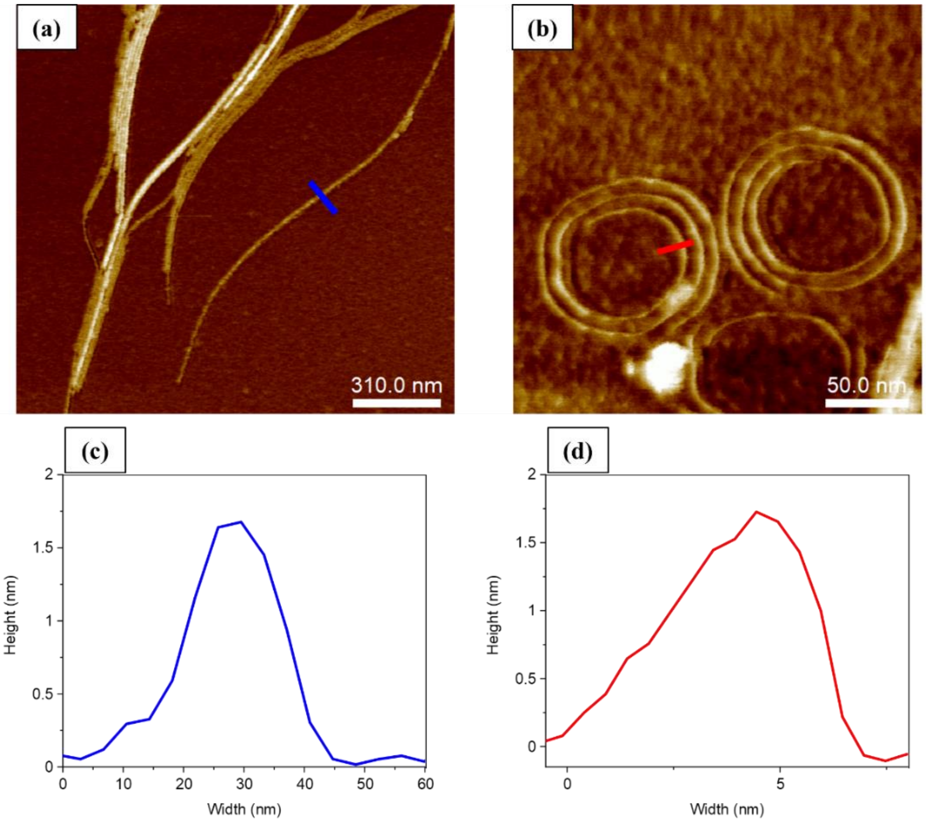

Figure S22. AFM images of the morphologies formed by the self-assembly of (a) **PDI-1** in THF/H_2O (30:70), and (b) **PDI-3** in THF/H_2O (50:50) at a concentration of 5 × 10^{-6} M. Height profiles along the corresponding colored lines are shown in (c) and (d), highlighting the curvature properties of **PDI-3** compared to **PDI-1**, where **PDI-3** fibers appear thicker on the outer side of the curves.

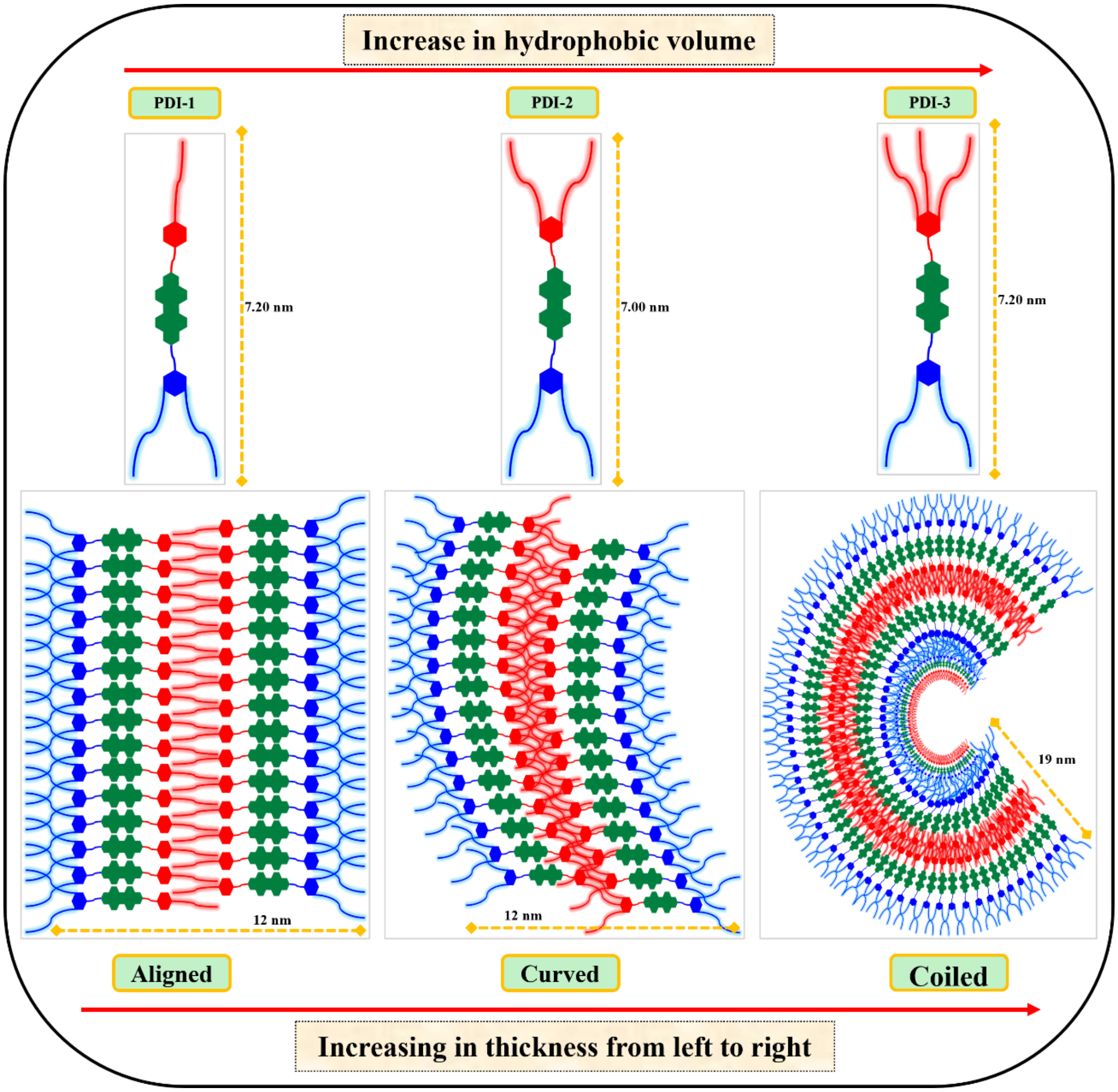

Figure S23. Overview of the proposed stacking mode in the self-assembly behaviour of PDIs, illustrating the increase in curvature of the self-assembled fibres from **PDI-1** to **PDI-3**. The schematic representation highlights the PDI core in green, the hydrophilic

Table S3. Summary of the estimated volumes of the hydrophobic and hydrophilic groups used in the synthesis of **PDI-1**, **PDI-2**, and **PDI-3** compounds.

	**Hydrophobic side (increasing)**			**Hydrophilic side (constant)**		
**PDI**	**(-CH_2-) Alkyl chain**	**(-CH_3)**	**Total Volume (nm^3)**	**(-CH_2-CH_2-O-) OEG groups**	**(-CH_3)**	**Total Volume (nm^3)**
**PDI-1**	**11**	**1**	**0.34 nm^3**	**16**	**1**	**1.088 nm^3**
**PDI-2**	**22**	**2**	**0.68 nm^3**	**16**	**1**	**1.088 nm^3**
**PDI-3**	**33**	**3**	**1.02 nm^3**	**16**	**1**	**1.088 nm^3**

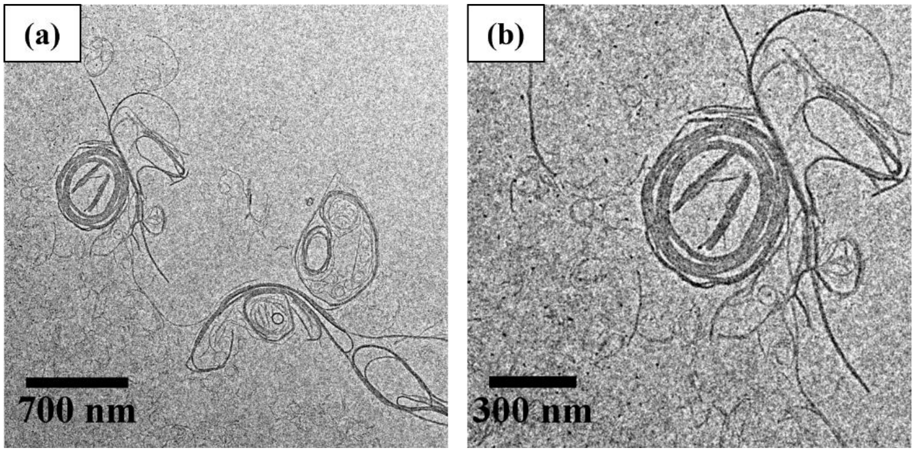

Figure S24. (a, b) Additional TEM images showing the coiled structures formed by the self-assembly of **PDI-3** in THF/H_2O (50:50) at a concentration of 5 × 10^{-6} M.

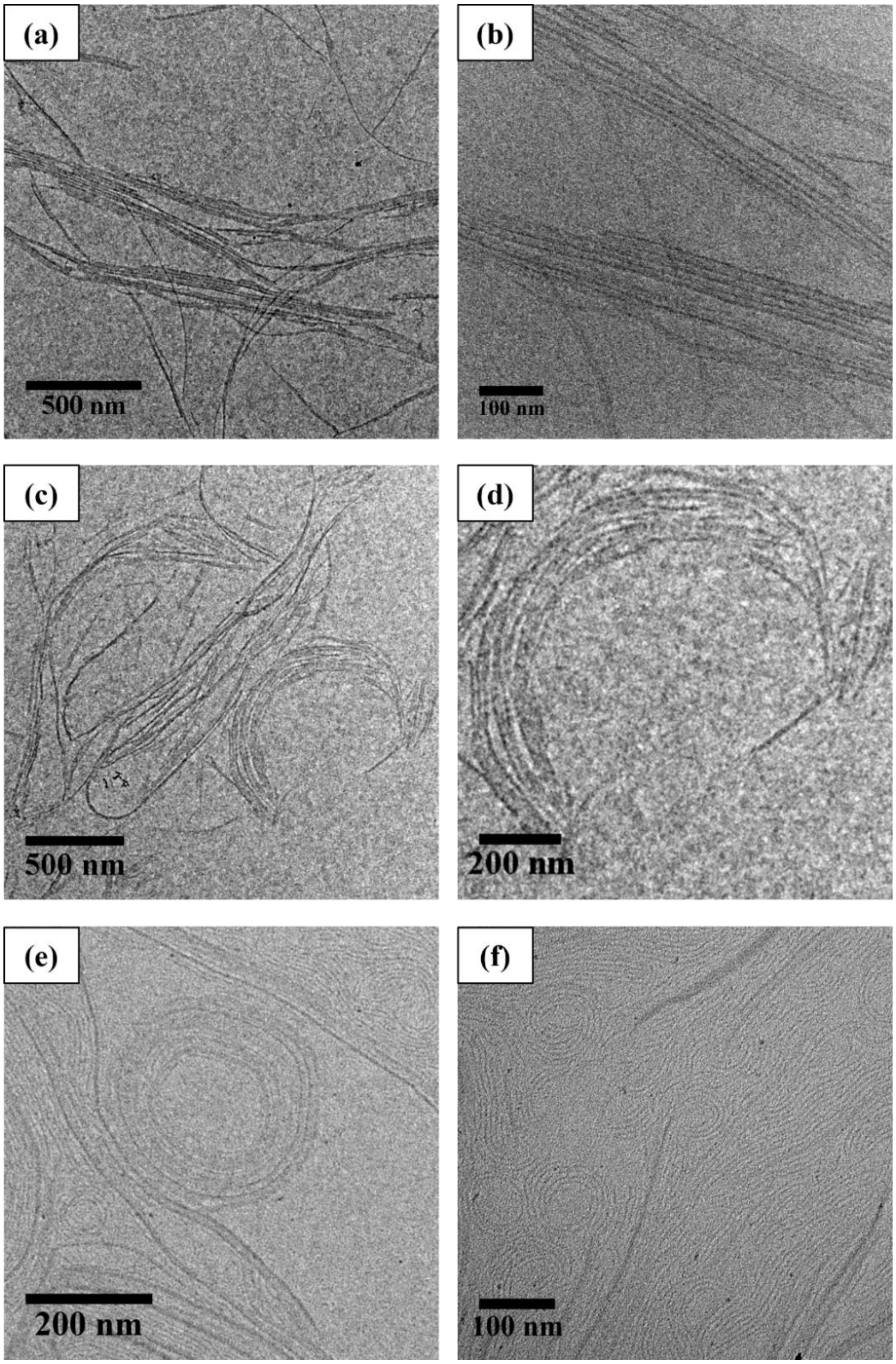

Figure S25. Additional TEM images at higher magnification showing the morphologies formed by the self-assembly of (a, b) **PDI-1** in THF/H_2O (30:70), (c, d) **PDI-2** in THF/H_2O (40:60), and (e, f) **PDI-3** in THF/H_2O (50:50) at a concentration of 5 × 10^{-6} M.

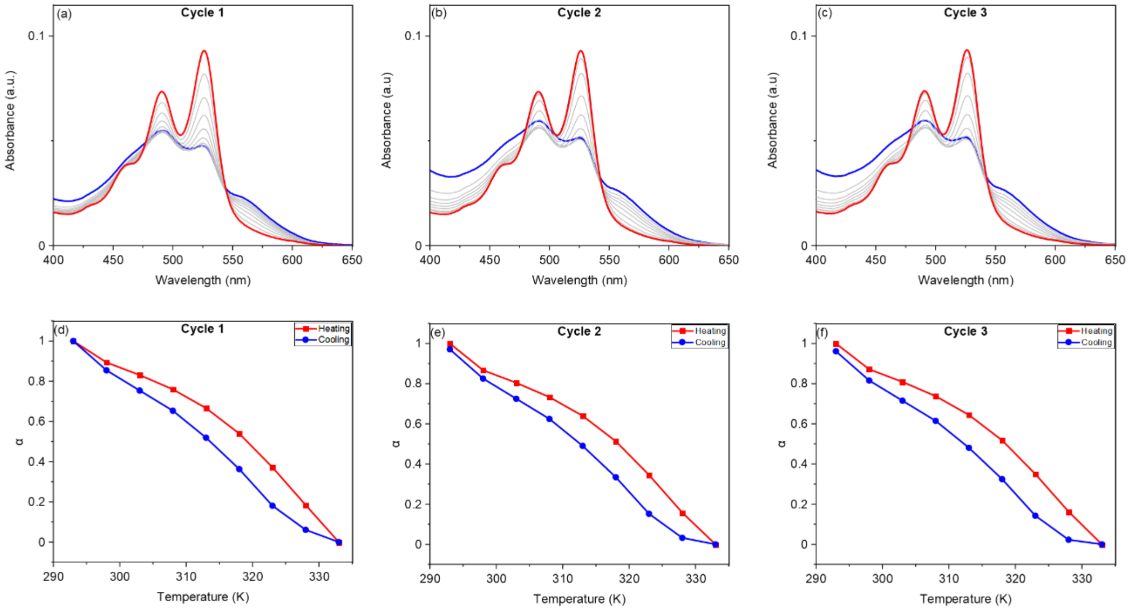

Figure S26. Changes in the UV/Vis absorbance spectra of **PDI-1** (a, b, c) recorded over three heating and cooling cycles from 293 K (blue profiles) to 333 K (red profiles) in 5 K intervals. The corresponding degree of aggregation (α) as a function of temperature for each cycle is shown in (d, e, f). Spectra were recorded in THF/H_2O (30:70) at a concentration of 5×10^{-6} M.

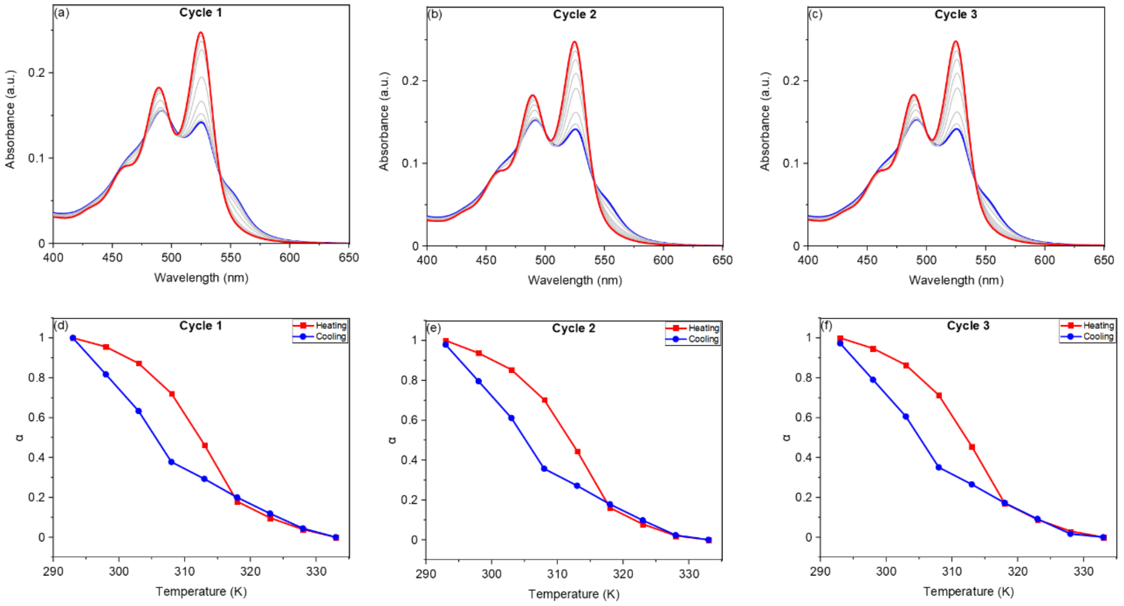

Figure S27. Changes in the UV/Vis absorbance spectra of **PDI-2** (a, b, c) recorded over three heating and cooling cycles from 293 K (blue profiles) to 333 K (red profiles) in 5 K intervals.

The corresponding degree of aggregation (α) as a function of temperature for each cycle is shown in (d, e, f). Spectra were recorded in THF/H_2O (40:60) at a concentration of 5 × 10^{-6} M.

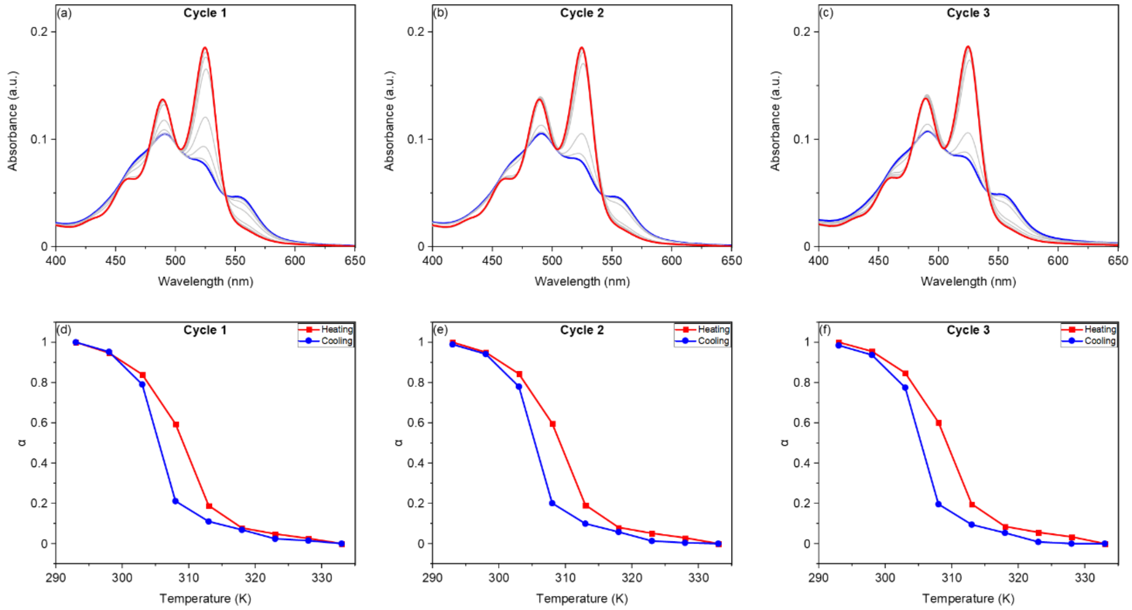

Figure S28. Changes in the UV/Vis absorbance spectra of **PDI-3** (a, b, c) recorded over three heating and cooling cycles from 293 K (blue profiles) to 333 K (red profiles) in 5 K intervals. The corresponding degree of aggregation (α) as a function of temperature for each cycle is shown in (d, e, f). Spectra were recorded in THF/H_2O (50:50) at a concentration of 5 × 10^{-6} M.

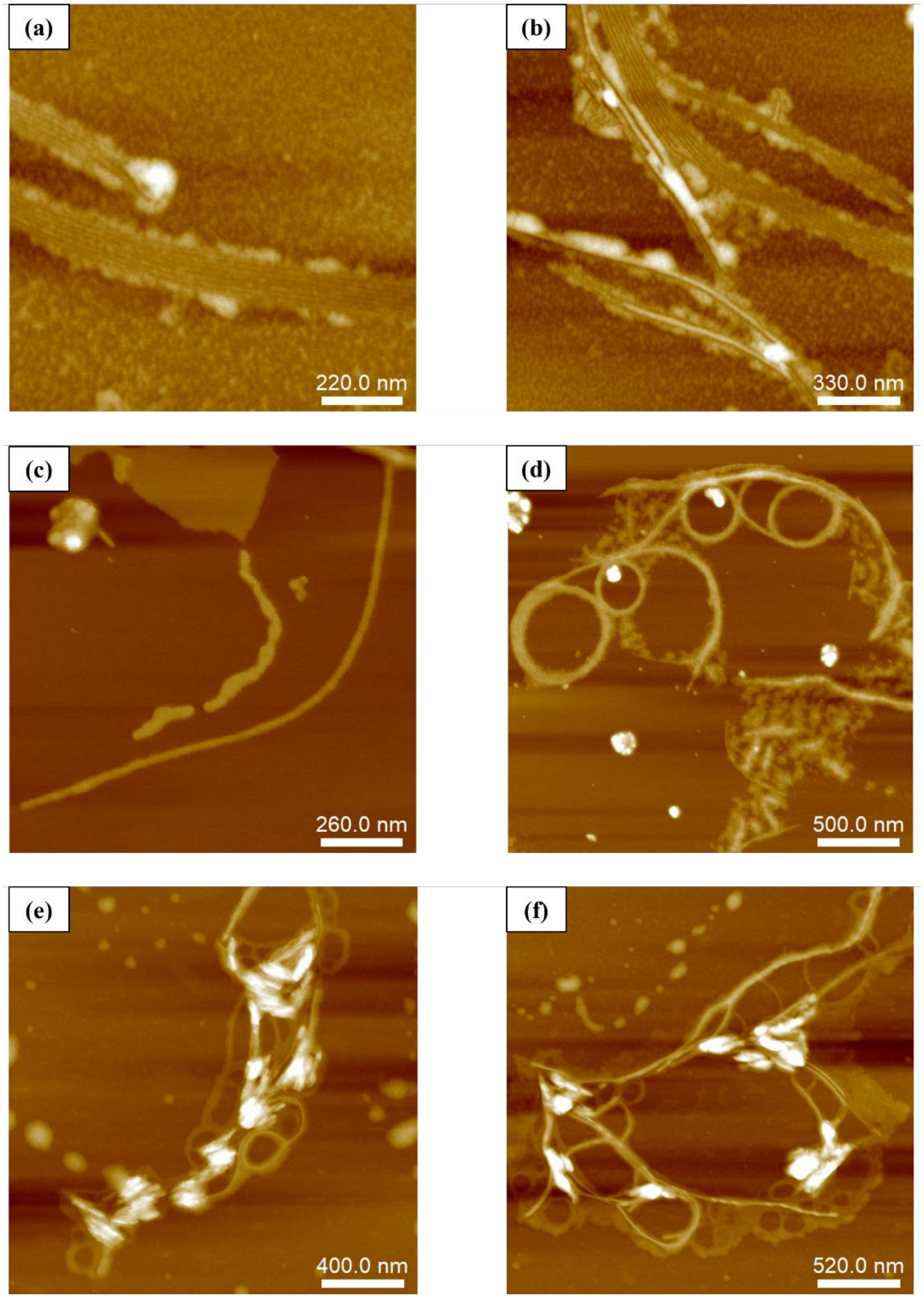

Figure S27. Additional AFM images of the morphologies formed by the self-assembly of (a, b) **PDI-1** in THF/H_2O (30:70), (c, d) **PDI-2** in THF/H_2O (40:60), and (e, f) **PDI-3** in THF/H_2O (50:50) at a concentration of 5×10^{-6} M. These images were recorded as part of time-dependent morphology measurements on PDI samples that had been prepared over six months earlier and stored on mica substrates, highlighting the stability of the formed self-assembled structures.

The temperature-dependent degree of aggregation, $\alpha_{(T)}$, was determined for each sample using Equation S1. $\text{Abs}_{(T)}$, $\text{Abs}_{(agg)}$, and $\text{Abs}_{(mon)}$ indicate the absorbance readings at a certain temperature, in completely aggregated and entirely monomeric forms, respectively.

Equation S1:

$$\alpha_{(T)} = \frac{\text{Abs}_{(T)} - \text{Abs}_{(mon)}}{\text{Abs}_{(agg)} - \text{Abs}_{(mon)}}$$

For isodesmic systems, data were fit to a sigmoidal curve which is characteristic of the isodesmic model, and the value of α corrected according to the predicted maximum value from fitting. Then the number-averaged degree of polymerisation, DP_n, was calculated using Equation S2.[3]

Equation S2:

$$DP_{n(T)} = \frac{1}{\sqrt{1 - \alpha_{(T)}}}$$

The equilibrium constant, K_e, was determined using Equation S3, where c_T represents the total concentration.

Equation S3:

$$DP_{n(T)} = \frac{1}{2} + \frac{1}{2}\sqrt{4K_{e(T)}c_T + 1}$$

For isodesmic systems, Van't Hoff plots were used to determine the changes in enthalpy, ΔH, and entropy, ΔS, values as described by Equation S4.

Equation S4:

$$\ln K_e = -\frac{\Delta H}{RT} + \frac{\Delta S}{R}$$

The change in Gibbs free energy, ΔG, was calculated using Equation S5.

Equation S5:

$$\Delta G = \Delta H - T\Delta S$$

The cooperative system was fitted to a model system proposed by Smulders et al.[4,5] The elongation and nucleation phases are described by Equations S6 and S7, respectively, with α_{sat} the fraction of aggregated molecules, T_e the elongation temperature, ΔH_e is the enthalpic change due to binding interactions during elongation, T is the absolute temperature in K, R the gas constant, and K_a the equilibrium constant.

Equation S6:

$$\alpha_{\mathrm{n}} = \alpha_{\mathrm{sat}}\left[1 - e^{\left(\frac{-\Delta H_{\mathrm{e}}}{R T_{\mathrm{e}}{}^{2}}(T - T_{\mathrm{e}})\right)}\right]$$

Equation S7:

$$\alpha_{\mathrm{n}} = K_{\mathrm{a}}{}^{\frac{1}{3}} e^{\left[\left(\frac{2}{3} K_{\mathrm{a}}{}^{-\frac{1}{3}}\right)\frac{\Delta H_{\mathrm{e}}}{R T_{\mathrm{e}}{}^{2}}(T - T_{\mathrm{e}})\right]}$$

The number-averaged degree of polymerization at the elongation temperature was calculated using Equation S8. At any other temperature, the degree of polymerization was determined by Equation S9.

Equation S8:

$$\langle N_{\mathrm{n}}(T_{\mathrm{e}})\rangle = \frac{1}{K_{\mathrm{a}}{}^{\frac{1}{3}}}$$

Equation S9:

$$\langle N_{\mathrm{n}}\rangle = \frac{1}{\sqrt{K_{\mathrm{a}}}}\frac{\alpha_{\mathrm{n}}}{\alpha_{\mathrm{sat}} - \alpha_{\mathrm{n}}}$$

4 References

[1] C. Jarrett-Wilkins, X. He, H. E. Symons, R. L. Harniman, C. F. J. Faul, I. Manners, *Chem. – A Eur. J.* **2018**, *24*, 15556.

[2] H. E. Symons, M. J. L. Hagemann, R. L. Harniman, C. F. J. Faul, *J. Mater. Chem. C* **2022**, *10*, 2828.

[3] G. A. Bhavsar, S. K. Asha, *Chem. - A Eur. J.* **2011**, *17*, 12646.

[4] M. M. J. Smulders, A. P. H. J. Schenning, E. W. Meijer, *J. Am. Chem. Soc.* **2008**, *130*, 606.

[5] M. M. J. Smulders, M. M. L. Nieuwenhuizen, T. F. A. De Greef, P. Van Der Schoot, A. P. H. J. Schenning, E. W. Meijer, *Chem. - A Eur. J.* **2010**, *16*, 362.
